# Structure-Based Training: A Training Method Aimed at Pixel Errors for a Correlation-Coefficient-Based Neural Network

**DOI:** 10.3390/s24206761

**Published:** 2024-10-21

**Authors:** Jun Su, Wei He, Yingguan Wang, Zhiyong Bu, Tiantian Zhang

**Affiliations:** 1 Shanghai Institute of Microsystem and Information Technology, Chinese Academy of Sciences, Shanghai 200050, China; j_su@mail.sim.ac.cn (J.S.);; 2 University of Chinese Academy of Sciences, Beijing 101408, China

**Keywords:** few-shot segmentation, pixel errors, shadow nodes, computer vision, bionic

## Abstract

In research on building a one-shot learning neural network without pre-training using mass data, the limitation on the information obtained from a single training sample downgrades the performance of the network. In order to improve performance and take full advantage of the support set, in this study, we design three kinds of shadow nodes and propose a structure-based training method for a correlation-coefficient-based neural network. This training strategy focuses on branches that are not activated or inactivated as expected. In contrast to existing networks that optimize the parameters using back-propagation, the training method proposed in this paper optimizes the structure of the correlation-coefficient-based network by correcting its pixel errors. For the shadow nodes and training process based on this strategy, the intersection over union (IOU) of a detected target increases by 4.83% in the experiments when using the Fashion-Mnist dataset, increases by 4.02% when using the Omniglot dataset, and increases by 3.89% when using the Cifar-10 dataset. The samples in category “7” wrongly classified as “1” decreased by 27.32% when using the Mnist dataset after training. This training strategy, along with shadow nodes, makes the correlation-coefficient-based network a more practical model and enables the network to develop during the accumulation of reliable samples, thus making it more suitable for simple target detection projects that collect samples over time. Moreover, the shadow nodes and training method proposed in this paper supplement the non-gradient-based parameter-gaining strategy. Additionally, it is a new attempt to explore the imitation of a human’s ability to learn a new pattern from a low number of references.

## 1. Introduction

In recent decades, one-shot or few-shot detection (OSD and FSD for short, respectively) and localization have seen broad application prospects. Currently, most OSD or FSD methods rely heavily on pre-training, which usually involves mass data and a large amount of computational resources. In order to alleviate the conflict between the cost of pre-training and the limitation of resources, as well as to mitigate the problem caused by the domain gap between the training sets and testing sets (source domain and target domain, more precisely), the authors in [1] proposed an approach to a non-pre-training-based one-shot network that could be constructed using only one training sample. Accordingly, specially designed neuron nodes and a correlation-coefficient-based structure, along with their calculation process, were designed in coordination with the model. 

Although this design enables the model to detect and locate simple targets without pre-training, it faces the problem that no existing training strategy could be applied to such a model. The gradient-based training method usually requires several back-propagations of errors before convergence, and the learning rate greatly interferes with the learning speed and the performance of the network. Most of all, the activation function of the correlation-coefficient-based model is non-differentiable; thus, the gradient cannot be computed. Therefore, a training strategy that works in correlation-coefficient-based networks is needed to realize the knowledge accumulating process.

Apart from gradient-based training strategies, which are the most widely used in existing models, bionic training strategies are also applied in the training processes of artificial neural networks. Evolutionary algorithms (EAs) can not only train the weights of connections between the neurons but also optimize the structure of the networks. However, EAs involve multiple networks and several generations, which require heavier computation.

In this paper, we propose a structure-based training strategy that adjusts the network’s structure according to the training samples and decreases the difference between segmented area and ground truth. Different from the gradient-based strategy that has been widely used, this structure-based strategy does not require gradient computing and focuses more on the activation of nodes, which equals the activation of branches in the structure. In contrast to EAs, this strategy does not require multiple generations of optimization. Through comparing the areas that reflect ground truth with those captured by the network, the wrongly activated nodes are recombined or separated to form new nodes and substructures and eventually trained to act correctly according to a targeted set of patterns. By coordinating with the training strategy, we designed three more kinds of shadow nodes that supplement the functions of existing neurons in a correlation-coefficient-based network. As far as we know, this is the first time a network has been trained with a purely structural strategy, and it is the first time a structure-based training strategy that works on a single sample has been proposed. This training strategy supplements the non-gradient-based parameter-gaining strategy that was introduced in [1]. Additionally, it contributes to the information accumulation progress for correlation-coefficient-based networks and, therefore, enables the network to develop while more samples are collected during its task, thus making the network more practical in the FSD of simple targets.

## 2. Related Works

The neural network (NN) model was first proposed by Warren MacCulloch and Walter Pitts in the 1940s [2]. However, not until the 1970s, when Paul Werbos came up with the back-propagation method [3], with Rumelhart popularizing it [4], did the NN gain its training method and open an important research field. Gradient-based back-propagation methods are still the main approach for NN training today. The YOLO series [5,6,7,8,9,10,11], for example, are trained using a gradient-based method. Over the years of studying NNs, researchers have come up with many gradient-based methods. Amongst these methods, gradient descent is a fundamental algorithm. It adjusts the weights of connections according to the gradient calculated with the chain rule. This strategy greatly reduces the computation cost. In order to improve the performance and convergence speed of the network, former researchers have proposed many strategies, such as stochastic gradient descent (SGD) [12], SGD with momentum (SGDM) [13], adaptive momentum estimation (Adam) [14], the second-order descent algorithm [15], etc. Furthermore, strategies such as the projection and rejection product (PR product) [16] and root mean square propagation (RMSProp) [17] have been introduced to address the vanishing gradient problem. Although gradient-based strategies are mature solutions to the network training problem, they face a fatal problem when applied to networks with non-differentiable activation functions.

Another approach to NN training is through evolutionary algorithms (EAs), which do not require gradient computation. In most cases, EAs can not only change the parameters but also optimize the structure of an NN. The NEAT algorithm [18], which is one of the foundational theories of correlation-coefficient-based networks, is a typical EA. NEAT encodes the networks into chromosomes with a direct coding strategy and produces its offspring by crossing over the parent chromosomes, followed by a mutation procedure. The networks are then filtered according to their fitness. Knowledge evolution (KE) [19] splits a network into a fit hypothesis and reset hypothesis and perturbs the reset hypothesis to evolve the fit hypothesis over generations. This approach enables the network to be trained on relatively small datasets and to learn a slim network with lower costs. Particle swarm optimization (PSO) [20,21] imitates foraging birds and inspires NN training strategies. The work in [22] takes advantage of the fast convergence of PSO and replaces the normally distributed variables with Gaussian random variables to enhance the local search problem of PSO. Additionally, it introduces fuzzy reasoning to structure learning to speed up the training process; multiple training strategies can be organized to improve the performance of NNs [23]. EA methods mainly involve multiple networks in an environment and require several generations of evolutions before convergence. Such requirements limit their application to FSD problems.

During the years of development of neural networks, researchers have found that the network structure plays an important role in the network’s performance. The YOLO series, for example, take advantage of the structure changes over generations. YOLO 3 [7] introduced darknet-53 to the network as the backbone, and YOLO 4 [8] added new modules, improving its average precision (AP) and frames per second (FPS) dramatically. YOLO 6 [9] redesigned the backbone and made further progress. Moreover, research on deep networks tend to apply mature backbones with pre-trained parameters to part of the structure, such as the networks proposed in [24,25], which apply ResNet [26] as the backbones. As suggested by Adam Gaier [27], networks with proper structure could work on random weights, emphasizing the importance of structure.

A correlation-coefficient-based network [1] was designed to imitate a human’s logic of learning a simple pattern and capturing the learned pattern. The network structure could be built on a single sample (which is called a construction sample in this paper) and could tell if the learned object is in the testing sample with a rough segmentation. While learning a pattern, the network records the pixels of lower-level features and their combinations that form higher-level features. When it comes to the testing phase, every neuron works like a simple metric-based network [23] that compares the testing picture block with the recorded pixels or combinations. As the feedforward and activation function is based on a correlation coefficient with a threshold of 0.8 [1] (as shown in Equation (1), which comes from a common standard for “highly correlated”), which is non-differentiable, gradient-based training methods could not be applied to the training procedure of a correlation-coefficient-based network. Moreover, EAs fail to meet the requirement of limiting the training samples to as few as possible (even one in most cases) and fail to make the training procedure direct and fit a similar logic to human learning patterns. When considering the physical sense of the parameters and branch-like structure of the correlation-coefficient-based model, a training strategy that handles the network structure is more plausible.

(1) $$y=\left\{\begin{array}{ll} 0, & \frac{\sum_{i=1}^{n}(P_{i}-\bar{P})(K_{i}-\bar{K})}{\sqrt{\sum_{i=1}^{n}{\left(P_{i}-\bar{P}\right)}^{2}\sum_{i=1}^{n}{\left(K_{i}-\bar{K}\right)}^{2}}}<threshold\\ 1, & \frac{\sum_{i=1}^{n}(P_{i}-\bar{P})(K_{i}-\bar{K})}{\sqrt{\sum_{i=1}^{n}{\left(P_{i}-\bar{P}\right)}^{2}\sum_{i=1}^{n}{\left(K_{i}-\bar{K}\right)}^{2}}}\geq threshold \end{array}\right.$$

In this paper, we propose three kinds of shadow nodes that supplement the function of the correlation-coefficient-based network. We also propose a structure-based training strategy for a correlation-coefficient-based network to address the knowledge accumulating problem. This paper is organized as follows: In Section 1, we give a brief introduction of this paper. Section 2 gives a brief review of the common training strategies of NNs and analyzes the characters needed by the training strategy applied to a correlation coefficient network. Section 3 shows the method; in Section 3.1 and Section 3.2, we put forward three types of shadow nodes that supplement the network function and support the training procedure. Section 3.3 proposes the structure-based training strategy, the results of which are shown in Section 4.

## 3. Materials and Methods

**Figure 1:**
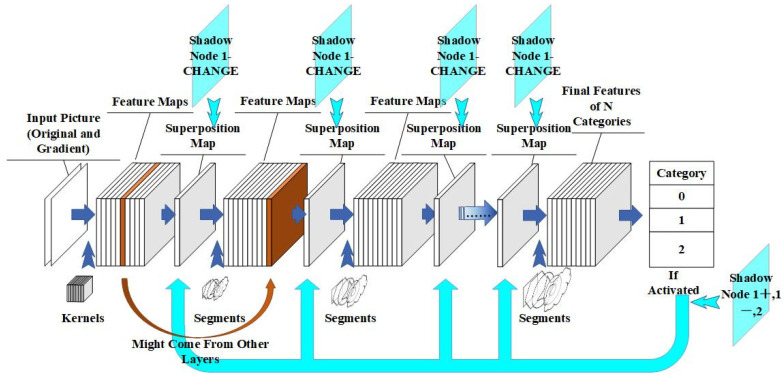
The way the shadow nodes function in the network structure. A shadow node 1-CHANGE is calculated in the feedforward process, and the other shadow nodes are calculated after the feedforward process.

The shadow nodes and training strategy proposed in this paper are supplements to the correlation-coefficient-based network proposed in [1] and enable the network to accumulate knowledge via a support set in the testing domain. The structure of the correlation-coefficient-based network serves a jigsaw-like logic that combines smaller features into larger ones. Therefore, the training logic focuses on two parts: (1) produce better nodes that interpret the target and handle the logical relationships between nodes; (2) optimize the network structure that represents the combinations and activation states of the nodes. Figure 1 shows how shadow nodes work in the network.

**Figure 2:**
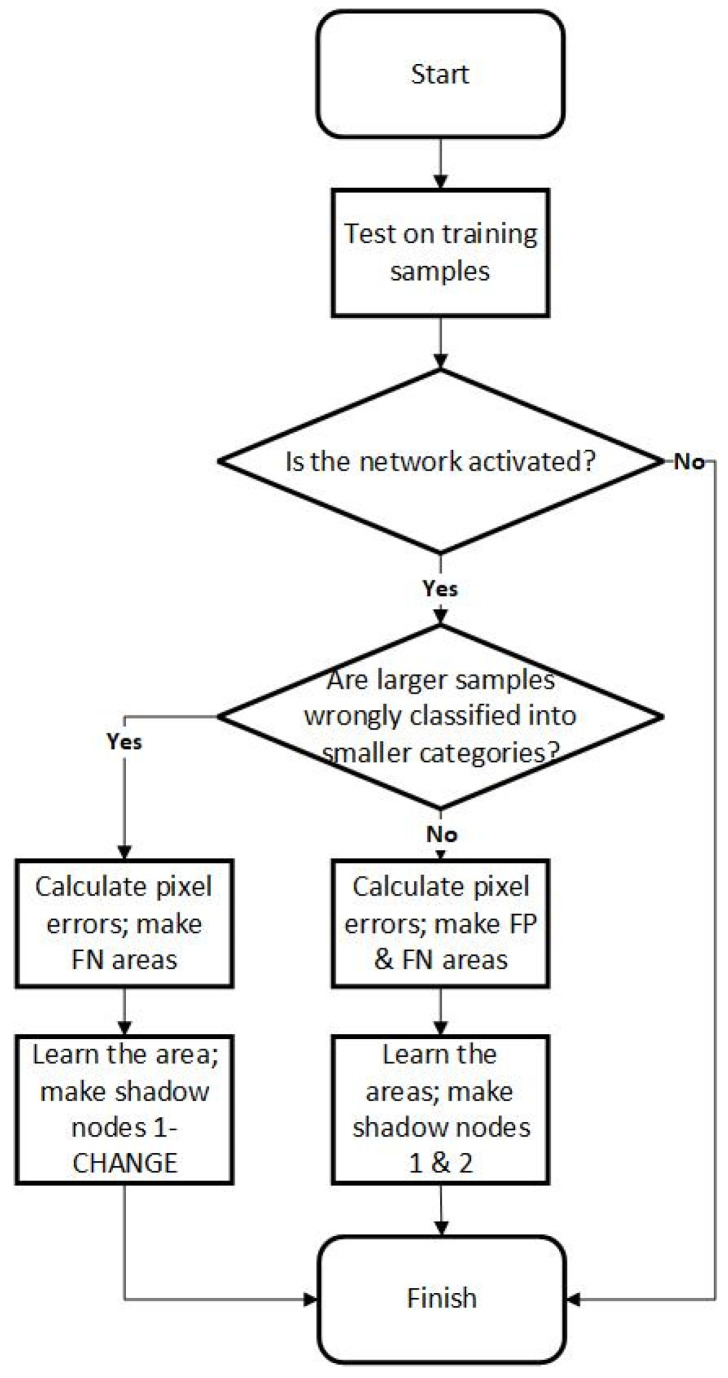
A flowchart of the training procedure.

As shown in Figure 1, the shadow nodes have two ways to interfere with the network. Shadow nodes 1+, 1-, and 2 work after the computing of the feedforward process is finished and only affect the segmentation result without changing the activation of a node in higher layers. A shadow node 1-CHANGE is computed in the feedforward phase and may interfere with the activation state of nodes in higher layers, even the final node. The training procedure is described in Figure 2.

### 3.1. Setting Up Shadow Nodes

In [1], we introduced two basic types of nodes, kernels, and segments and one kind of shadow node that performs “or” logic. Kernels and segments form the network structure, whereas the shadow nodes deal with the logical relationship among nodes. As the network is calculated using layers and a node should be calculated after its child nodes are computed, the level of a shadow node should be assigned according to a different rule from that obeyed by basic nodes. Moreover, the center of a node influences its detecting area. Therefore, the center is decided according to the need of shadow nodes. The shadow nodes imitate a human’s short-term memory, which temporarily corrects the deviation between detected objects and the learned patterns. The activated kernels and segments are recorded in feature maps, and the following shadow nodes are supposed to re-edit the feature map.

**Figure 3:**
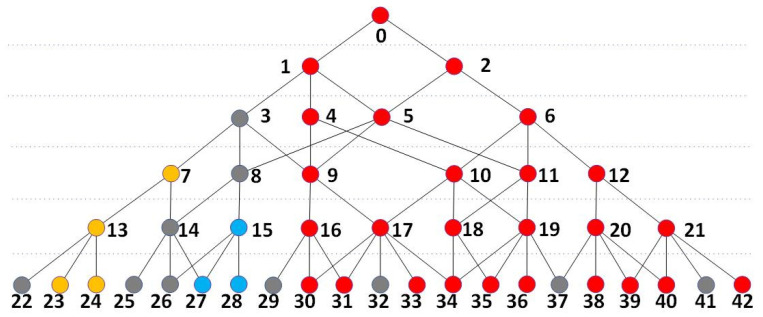
A schematic of a correlation-coefficient-based network structure. The gray nodes are inactive nodes, and the yellow, red, and blue nodes are activated nodes in different branches.

In order to describe the training strategy more conveniently and precisely, we first need to define the nodes that contribute to the activation of the network. As shown in Figure 3, if an activated node (A) is chosen by its parent node and this parent node is also activated, node A is a node that contributes to its parent node. The correlation coefficient network has a tree-like structure, and if a branch starts from any node that is higher than the kernel layer, then we call every activated node in this branch to contribute to the branch. If the final node is activated or the root of the tree-like structure is activated, then any activated node in the structure contributes to the activation of the network. 

The gray nodes in Figure 3 represent inactive nodes or nodes that fail to be anchored. The nodes in red, blue, and yellow are activated nodes. The final node, which is numbered 0, is activated, meaning the network structure is activated, and all the red nodes contribute to the activation of this network structure. As nodes 27 and 28 are activated child nodes of their parent node 15, which is also activated, nodes 27 and 28 contribute to their parent node. Similarly, nodes 23 and 24 contribute to their parent node 13. For any branch that starts from an activated node, $N_{top}$, every activated node in this branch except for $N_{top}$ contributes to this branch.

**Figure 4:**
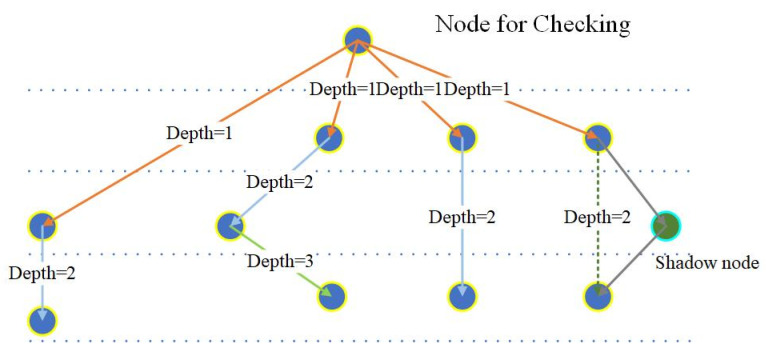
Tracing depth of nodes. The orange lines indicate depth = 1, the blue lines indicate depth = 2, and the green lines indicate depth = 3.

The shadow nodes introduced in this paper do not occupy the tracing depth, which is shown in Figure 4.

The depth of a node starts from the node that needs to be traced to obtain its lower nodes that contribute to it or to obtain the recorded nodes in the templates that construct the network structure. The depth does not necessarily equal the layer difference. The depth of the segments and kernels are calculated by their ranks, which are counted by their fore-parents below the start node. When there are shadow nodes (proposed in this paper), they are simply skipped and do not contribute to the rank of the lower nodes in this branch.

#### 3.1.1. Conditionally Activated Shadow Nodes (Positive Term)

As the name suggests, this kind of shadow node only activates with additional conditions. The conditionally activated shadow node in the positive term needs a trigger node, which is denoted as $T_{P1+}$, which enables the other child nodes to be activated. It can be described as “and” logic.

**Figure 5:**
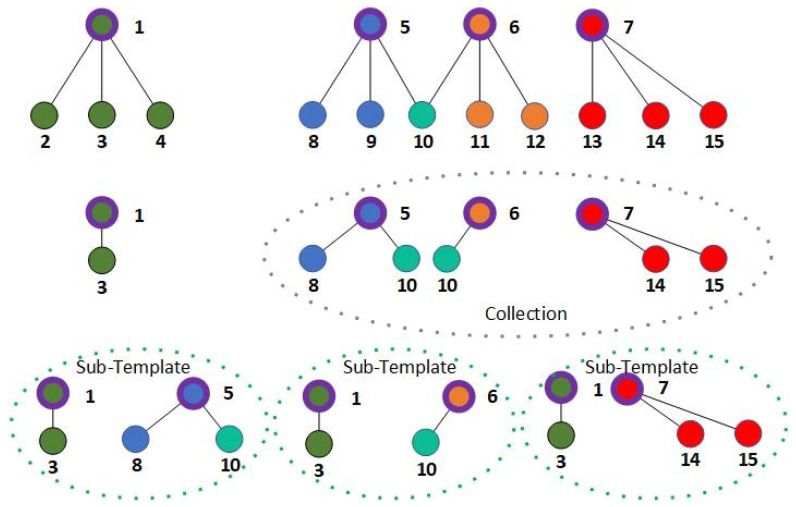
The construction of the structure of a shadow node 1+. The nodes with thicker borders are the parent nodes of the nodes in the same color with thinner borders. Node 10 has two parent nodes and is marked in a color different from both parent nodes.

When producing a shadow node, $S_{P1+}$, in this type, the child nodes, $C_{P1+}$, apart from the trigger node, are put into a collection, and their parent nodes, $P_{P1+}$, are also recorded, respectively. For every parent node $P_{P1+}$ for which the child nodes are recorded, the collection produces a sub-template, including the coordinates of the trigger node, $T_{P1+}$, child node(s), $C_{P1+Sub}$, and the parent node, $P_{P1+Sub}$. The structure is shown in Figure 5.

As shown in Figure 5, nodes 8, 10, 14, and 15 are chosen to produce a shadow node, 1+ $S_{P1+}$, and nodes 5, 6, and 7 are their parent nodes, $P_{P1+}$. Meanwhile, node 3 is chosen to be the trigger node, $T_{P1+}$, and node 1 is its parent node. After the needed nodes are chosen, child nodes 8, 10, and 14 (which are denoted as $C_{P1+}$) and their parent nodes are put in a collection. For each parent node, a sub-template is made, which includes a $P_{P1+Sub}$ with the corresponding $C_{P1+Sub}$. The trigger node, $T_{P1+}$, and its parent node are also added to enable the anchoring procedure in each sub-template.

Although the parent nodes stay in higher layers than the child nodes, they are recorded equally with the trigger node and child nodes involved in the shadow node to simplify the anchoring and calculating procedure. Since node $S_{P1+}$ deals with nodes that are taken into the network structure, it needs to decide whether the child nodes contribute to their parent nodes, $P_{P1+}$. As the shadow node has to be calculated after the parent node of the child nodes is calculated, the layer of this kind of shadow node is set to the next layer of the highest parent node, $P_{highest}$. The center of the shadow node is set to the position of the trigger node, $T_{P1+}$. In the rest of the paper, this kind of shadow node is called a shadow node 1+.

#### 3.1.2. Conditionally Activated Shadow Nodes (Negative Term)

Resembling the positive term, the conditionally activated shadow node in the negative term requires a trigger node, $T_{P1\text{-}}$. In contrast to the positive term, where the activation of child nodes in a collection is restricted by the trigger, the activation of the trigger node in the negative term is restricted by the nodes in the collection. 

Child nodes, $C_{P1\text{-}}$, in the collection and the trigger node, $T_{P1\text{-}}$, are supposed to be those that contribute to the activation of their parent node, $P_{P1\text{-}}$; thus, the layer of this shadow node, $S_{P1\text{-}}$, is set to the next layer of the parent node of the nodes in the collection. Moreover, the center of the shadow node is recorded in the same way as the trigger node, $T_{P1\text{-}}$, to simplify the calculation. In the rest of the paper, this kind of shadow node is called a shadow node 1-.

Resembling the structure of a shadow node 1-, a variant called a shadow node 1-CHANGE is also produced. The layer of a shadow node 1-CHANGE, which is denoted as $S_{P1\text{-}V}$, is set to the next layer of the highest parent nodes.

#### 3.1.3. Jointly Activated Shadow Node

This type of node is designed to make up for activated areas that are not included in the final structure. Different from basic nodes that work in the feedforward procedure, these kinds of nodes work in the back–forward process that traces activated nodes to segment the target area; therefore, producing a jointly activated shadow node will not influence the activation state of nodes when calculating the network. A jointly activated node consists of a trigger node, $T_{P2}$, and a collection of nodes, $C_{2}$, for which the parent nodes are not taken into account. When the trigger node, $T_{P2}$, is activated and contributes to the network, and the collection is activated even though it does not contribute to the network in the feedforward phase, the area covered by them will be added into the segmentation result if $T_{P2}$ and $C_{2}$ are anchored and then activate the shadow node successfully. 

It can be inferred that the structure of the “collection” has a function that resembles a segment. However, a formal segment is not produced; instead, the child nodes in the collection are recorded equally with the trigger node. As the activation function of the segments requires a certain length of the list containing child nodes, and a list with merely two nodes (the trigger node, $T_{P2}$, and the formal segment representing the collection) is obviously not a reliable template; recording them together is a good solution to the anchoring and calculation problem. In the rest of this paper, this kind of shadow node is referred to as shadow node 2.

### 3.2. Computing the Shadow Nodes

In [1], we proposed the computing rule of segments, which could be summarized as anchoring and calculating. In the anchoring process, the collected nodes that are activated in the sliding window are selected and paired with those recorded as child nodes in a segment. In the calculating process, the positional relationships between the nodes are defined by their distances and the angles between the half-lines connecting neighboring nodes in sequence. Furthermore, a checking procedure that filters the activated feature by its rotation, child nodes’ connectivity, and the locations of nodes with a tracing depth of 2 is introduced. The anchoring and calculating processes are not independent, as the traversal in the anchoring process stops when the calculation result reaches a threshold. Therefore, an activated node’s child nodes are also anchored, while the anchored nodes do not necessarily activate a parent node. The anchoring and calculating process is denoted as $F_{act\&anc}\left(w,k\right)$, where w stands for nodes collected in the window, and k stands for the template recorded in shadow nodes.

The calculation process of the shadow nodes obeys a similar rule to the segments. The nodes with the same IDs as the nodes needed by the shadow node that are collected in a window are first fast-filtered, and then anchored, calculated, and checked. The purpose of fast filtering is to examine whether the collected nodes can activate their parent nodes and decide whether to skip this window to speed up the calculating process.

The fast-filtering algorithm is denoted as $F_{ff}\left(w,k\right)$, where w represents the activated nodes collected in the sliding window on a feature map with the same IDs as those of the nodes in the template, and k represents the nodes recorded in the template of a segment. This function is applied in computing the shadow nodes. Another function $F_{absence}\left(w,k\right)$ is also introduced to supplement the fast-filtering procedure in shadow nodes with triggers. The function reflects if the collected nodes, w, are all missing compared to the template k. 

#### 3.2.1. Calculating a Shadow Node 1+

**Figure 6:**
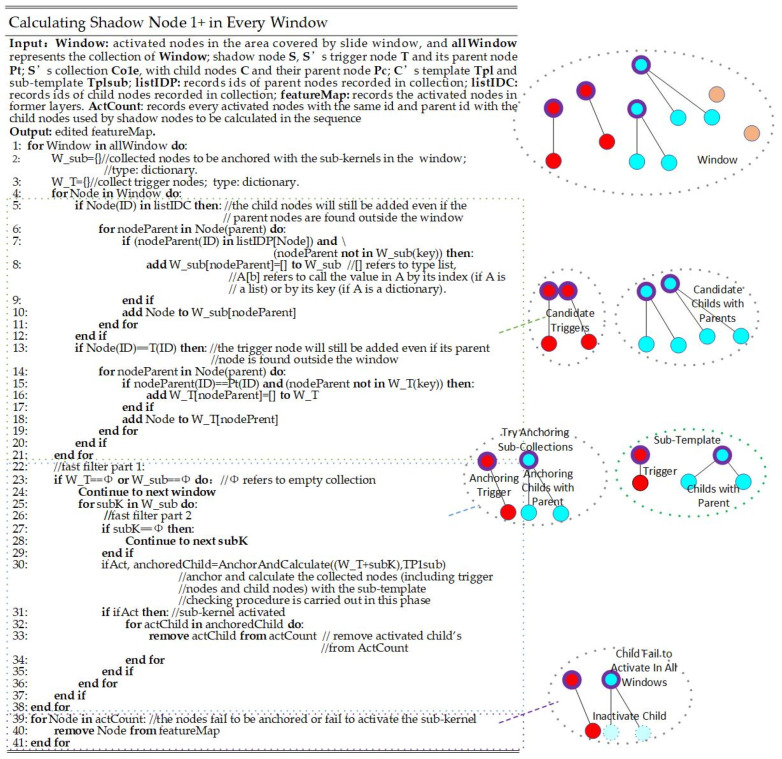
The pseudocode of the computing procedure of a shadow node 1+. The red nodes are trigger nodes and their parent nodes (with thicker borders), and the blue nodes are the child nodes in the collection and their parent nodes (with thicker borders).

The main purpose of a shadow node 1+ is to leave out the areas that are activated but not included in the ground truth. Thus, the calculation focuses on activated nodes that contribute to the network structure. In order to ensure the nodes are contributing to parent nodes, the activated parents that are recorded in the shadow node as a template are collected when the window slides throughout the picture. For a shadow node 1+ $S_{P1+}$, all the activated nodes with the same IDs as those nodes, as well as their parent nodes recorded in the template of $S_{P1+}$, will be recorded. Then, the needed child nodes are selected and listed separately with their parent node. For every group, we try to anchor the gathered nodes with those recorded in every sub-template. The details are shown in Figure 6.

Figure 6 shows the pseudocode of the computing procedure of a shadow node 1+. Yellow nodes are nodes for which the IDs or parent IDs are not included in node S. The red nodes are trigger nodes (with thinner borders) and their parent nodes (with thicker borders), while the blue nodes are child nodes (with thinner borders) and their parent nodes (with thicker borders). The sub-template marked in Figure 6 refers to the recorded template in node S, and the set of nodes on the left of the sub-template is one of the sets that try to anchor with the sub-template during traversal.

Denote the anchoring and calculating process of an ordinary segment as $F_{act\&anc}\left(w,k\right)$; the anchoring and calculating could be described as per Equation (2).

(2) $$F_{P1+}=F_{act\&anc}\left(w_{sub+t},k_{sub+t}\right)$$

$k_{sub+t}$ refers to a single sub-template, including the trigger node, and $w_{sub+t}$ refers to nodes collected from the sliding window according to the sub-template and trigger node.

In ordinary shadow nodes, the checking procedure includes three parts: an absence check $F_{check-missing}\left(a,k\right)$, rotation check $F_{check-rotate}\left(a,k\right)$, and connectivity check $F_{check-conn}\left(a,k\right)$. The rotation and connectivity check parts are the same as those in ordinary segments. The absence check, however, is different from that of an ordinary segment. In ordinary segments, if the missing nodes found in a window occupy a certain importance (which is decided by its area), then the segments that depend on this set of anchored nodes are inactivated. In the case of a shadow node 1+, although the computing will be skipped if no child nodes are collected for a sub-template, which is also reflected in fast filter procedures, the absence of partial child nodes will not influence the activation of the sub-template. The checking algorithm for every sub-template can be stated as per Equations (3) and (4):

(3) $$flag\left(sub-template\right)=F_{check-all-abs}\left(a_{tr},k_{tr}\right)\cdot F_{checkP1}\left(sub-template\right)$$

(4) $$F_{checkP1}\left(sub-template\right)=F_{check-all-abs}\left(a_{c,subi},k_{c,subi}\right)\cdot F_{check-rotate}\left(a_{t,subi},k_{t,subi}\right)\cdot F_{check-conn}\left(a_{t,subi},k_{t,subi}\right)$$

The function $F_{check-all-abs}\left(w,k\right)$ estimates if the nodes trying to be anchored in a single step in the traversal (denoted as a) are all missing compared to the kernel, k. Subscript tr represents the trigger found and needed, while the subscript c represents the child nodes in every sub-template. The subscript t represents the nodes in the sub-template along with the trigger. For every sub-template, flag=1 indicates that the nodes pass the check and the sub-template is activated.

The fast filter progress is also different from that when calculating the segments. Apparently, if a trigger node is missing, the child nodes in this window will not be activated. Moreover, if the child node is not activated at all, it will be impossible to activate it after the calculation of the node, S. Define the algorithm that estimates if the nodes found in window w are all missing compared to the kernel k as $F_{ff-absence}\left(w,k\right)$; then, the fast-filtering process for every sub-template can be denoted as per Equation (5).

(5) $$flag\left(sub-template\right)=F_{ff-absence}\left(w_{T},k_{T}\right)\cdot F_{ff-absence}\left(w_{c},k_{c}\right)$$

w represents the nodes collected in the sliding window according to the sub-template, while k represents the sub-template. Subscript T stands for the trigger node, and the subscript c stands for the child nodes in the sub-template. flag=1 indicates that the nodes pass the fast-filtering procedure, and the computation will be skipped if flag=0.

The child nodes that are not successfully anchored or defined as being activated will not be set to inactivate immediately. Only after the window has scanned the whole picture and a certain child node is not activated in any case will it be set to inactivated.

#### 3.2.2. Calculating a Shadow Node 1-

**Figure 7:**
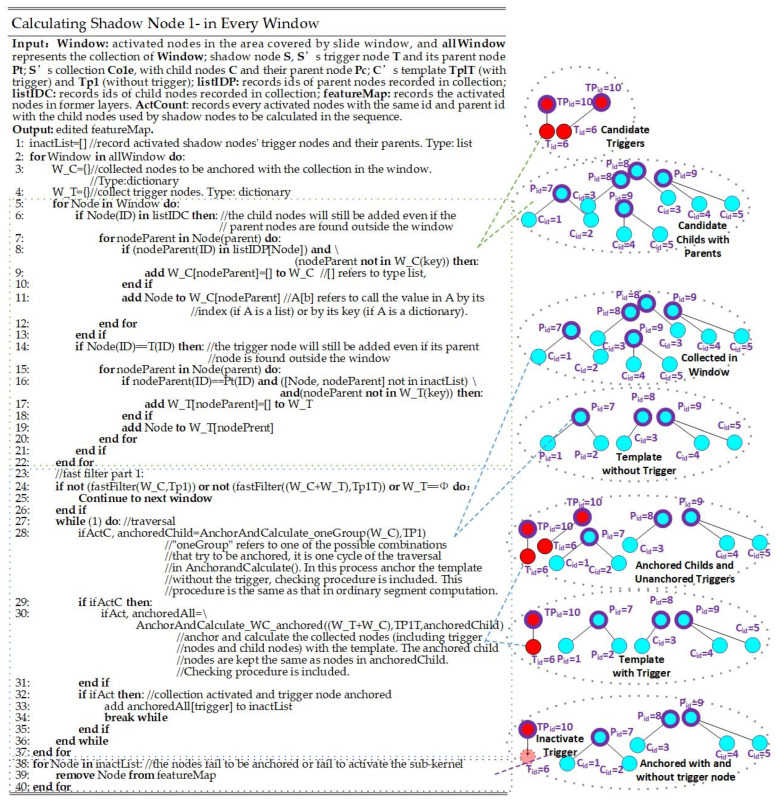
The pseudocode of the computing procedure of a shadow node 1-. The red nodes are trigger nodes and their parent nodes (with thicker borders), and the blue nodes are the child nodes in the collection and their parent nodes (with thicker borders).

A shadow node 1-, $S_{P1\text{-}}$, can be regarded as a combination of a segment and a trigger node, $T_{P1\text{-}}$, although no formal segment is produced. At the same time, $S_{P1\text{-}}$ represents a larger segment, including the nodes in a collection and the trigger node. Like in the procedure for a shadow node 1+, all activated nodes with the same ID as the parent nodes of trigger nodes and child nodes are collected and recorded. Then, the window slides to collect the trigger node and child nodes with the same parent nodes as those recorded in the shadow node, $S_{P1\text{-}}$. After that, the same anchoring and calculating procedure is run on the collected child nodes for the collection. If the collection is activated, choose an activated node with the same ID as the trigger node and try anchoring it with the collection added to the trigger node. The details are shown in Figure 7.

As shown in Figure 7, the trigger nodes (with thinner borders) and their parent nodes (with thicker borders) are marked in red, and the child nodes (with thinner borders) and their parent nodes (with thicker borders) are marked in blue. It can be seen that the anchoring and calculating process is carried out twice. The first time is to anchor the child nodes in the collection without the trigger node. After the collection is anchored, try anchoring the template again (trigger node included) with the fixed child nodes. If both templates are anchored successfully, and both templates are activated, the trigger node should be set to inactivated.

The fast-filtering algorithm of the ordinary segments is denoted as $F_{ff}\left(w,k\right)$, where w represents the activated nodes collected in the feature map, and k represents the nodes recorded in the template of a segment. The template in a shadow node 1- is treated as two templates: (1) a template of the nodes in the collection, denoted as $k_{c}$, which resembles the template of a segment without a formal segment; (2) a template including the nodes in the collection and the trigger node, denoted as $k_{t}$. The nodes collected in the window according to the IDs of the nodes and their parent nodes in the two templates are denoted as $w_{c}$ and $w_{t}$, respectively. The fast-filtering algorithm is shown in Equation (6).

(6) $$flag\left(template\right)=F_{ff}\left(w_{c},k_{c}\right)\cdot F_{ff}\left(w_{t},k_{t}\right)$$

The anchoring algorithm is shown in Equation (7).

(7) $$F_{P1\text{-}}=F_{act\&anc}\left(w_{c},k_{c}\right)\cdot \;F_{act\&anc}\left(w_{t},k_{t}\right)$$

We denote the check process in ordinary segments as $F_{check}\left(w,k\right)$, so the checking process of the shadow node is described in Equation (8).

(8) $$F_{checkP-}=F_{check}\left(w_{c},k_{c}\right)\cdot \;F_{check}\left(w_{t},k_{t}\right)$$

**Figure 8:**
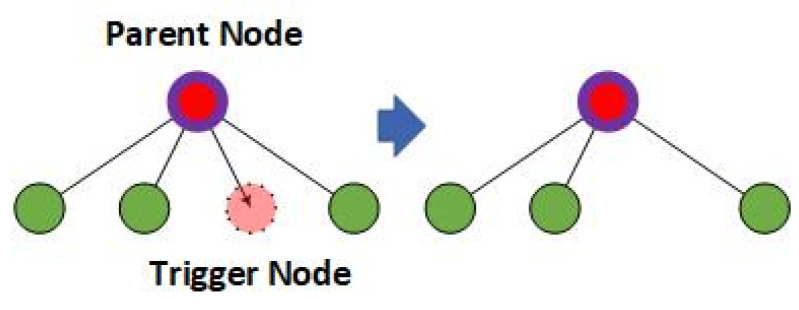
The parent node of the trigger node is calculated without the inactivated trigger node. The pink node stands for the node inactivated in the calculation. The red nodes are the re-calculated parent nodes (before and after re-calculation), and the green nodes are their child nodes that remain unchanged. The pink node is the inactivated node.

For a shadow node 1-CHANGE, the calculating sequence is carried out during the feedforward progress rather than after the final node is computed. The anchoring and calculating procedure are the same as that of the original shadow node 1-, and after the trigger nodes are set to inactivated, their parent nodes are computed again without those inactivated trigger nodes, as shown in Figure 8.

As shown in Figure 8, if a trigger node of a shadow node 1-CHANGE is inactivated, its parent node will be calculated again with anchored pairs of nodes fixed and with the trigger node excluded. Therefore, the activation of a shadow node 1-CHANGE may influence the activation of nodes in a higher layer and eventually the final node.

#### 3.2.3. Calculating a Shadow Node 2

A shadow node 2 shares a resembling calculation logic with that of a shadow node 1-. The main differences between a node 1- and node 2 are that the trigger node enables the collection to be added rather than inactivated by activated collection, and the child nodes in the collection of a node 1- have to contribute to the network structure, while those in a node 2 do not have to contribute to the activation of the whole structure. 

After the calculation of nodes in the highest layer, a shadow node 2 is calculated to make up for the incomplete areas. When anchoring the template, the trigger node and the corresponding node in the template is treated as a pair of anchored nodes.

### 3.3. Structure-Based Training Strategy of the Correlation-Coefficient-Based Network

The strategy promoted in this paper includes two types of training algorithms. The first one aims to improve segmentation accuracy and IOU, and the second one deals with classification, and an object’s area is contained by another object of a different type. The precondition is that the final node of the network structure has to be activated; thus, the training does not improve the possibility of an object being captured.

The shadow nodes proposed in this paper enable the network to optimize its segmentation results. Segmentation could be treated as a binary classification task. Unlike the existing NN, the error is reflected by a certain number, and the error of a correlation-coefficient-based network is reflected by a map. The first step is to make a map that includes the pixels that are misclassified.

The misclassified pixels could be classified into two kinds: false positive (FP) and false negative (FN). The segmentation result is computed via back-tracing the nodes that contribute to the structure all the way down to the kernels. When comparing the segmentation result to the efficient map provided for the training, the FP and FN pixels could be collected and written on a map according to their actual coordinates. The training process is based on the map.

The calculation of FP and FN areas requires segmentation results, which means that the final node has to be activated. This is the reason why the training procedure cannot be carried out on unsuccessfully activated networks.

#### 3.3.1. Training on FP Area

**Figure 9:**
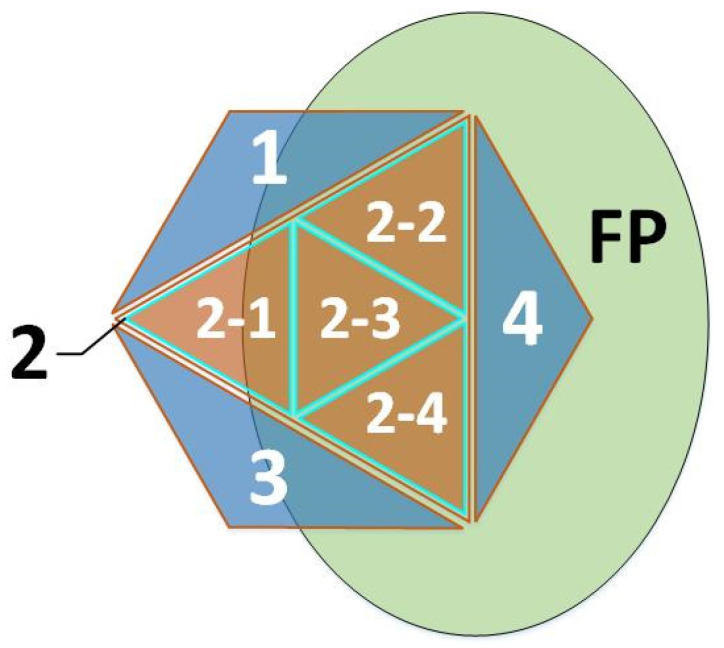
Find the highest nodes of the branches that totally fall into the FP area. The nodes 2-1, 2-2, 2-3, and 2-4 stand for the child nodes of node 2.

Apparently, the FP pixels are covered by activated feature nodes that are supposed to be inactivated; thus, the goal is to elide these nodes. Therefore, the corresponding branch (or fore-parent nodes in the higher layer) should be anchored with the FP areas. Find the highest nodes of the activated branches that totally fall into the FP area (Figure 9). 

**Figure 10:**
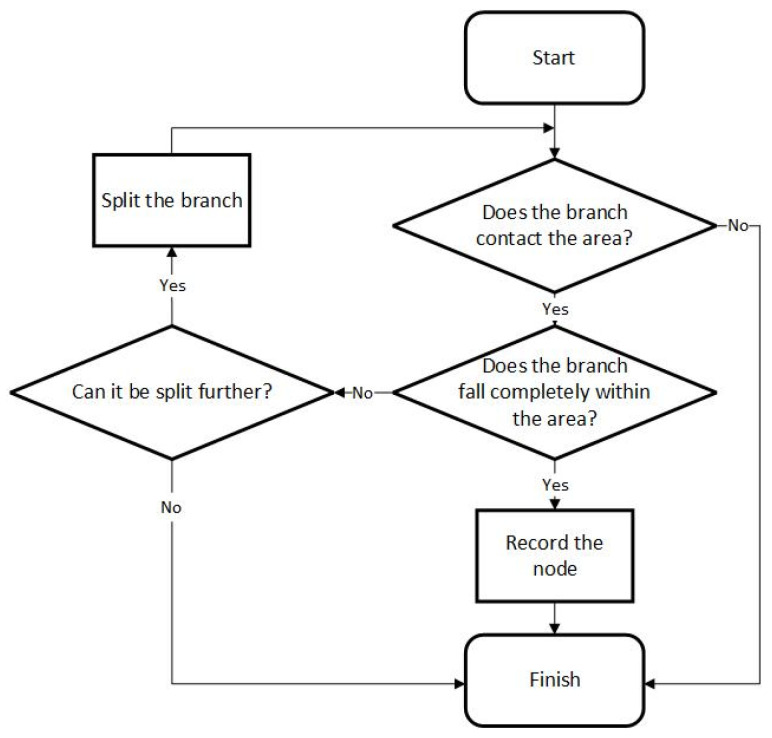
A flowchart of splitting the features and finding the highest nodes of the branches falling completely within the FP area.

The segments are traced to obtain the actual area they cover. If a segment partially falls into the FP area, then every node that contributes to it should be traced, too, to check if it totally falls into the area. If so, the nodes, along with their parent node, are recorded. In Figure 9, for example, segment 4 falls into the FP area, and then segment 4 and its parent node are recorded without further tracing. Segment 2 partially falls into the FP area, so the child nodes should be traced. It can be seen that segments 4, 2-2, 2-3, and 2-4 fall into the FP area, and segments 1, 2-1, and 3 need further tracing. If the segments are tracked down to the kernels, which are the basic units in the network structure, and the kernels are not fully included in the FP area, then they will be ignored. Therefore, this strategy has a one-pixel-wide basic error if the kernels do not fit in the FP areas and the centers happen to fall into the FP area or if the centers of the kernels fall out of the PF area but partial pixels reach into the FP area. The procedure is shown in Figure 10.

**Figure 11:**
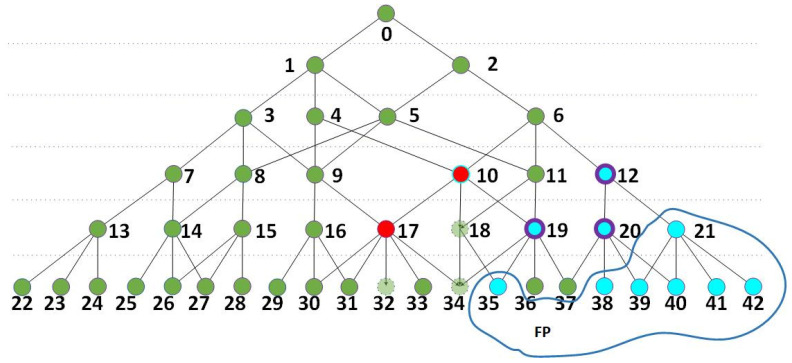
The procedure of building a shadow node 1+. The green nodes are basic nodes that form the network structure, and the light green nodes are inactive nodes in the testing phase. The blue nodes are the child nodes and their parent nodes (with thicker borders) that form the collection, and the red nodes are the trigger node and its parent node (with thicker borders). The child nodes that fall in the FP areas are marked with a blue circle.

As shown in Figure 11, the blue nodes with thinner borders (nodes 35, 38, 39, 40, 41, 42, and 21) fall into the FP area. Amongst the nodes above, nodes 35, 38, 39, 40, and 21 are the highest nodes in an activated branch and, thus, are recorded as child nodes. Their parent nodes, $P_{P1+}$—the blue nodes with a thicker border, node 19 (parent of 35), node 20 (parent of 38, 39, and 40), and node 12 (parent node of 21)—are recorded along with the child nodes.

**Figure 12:**
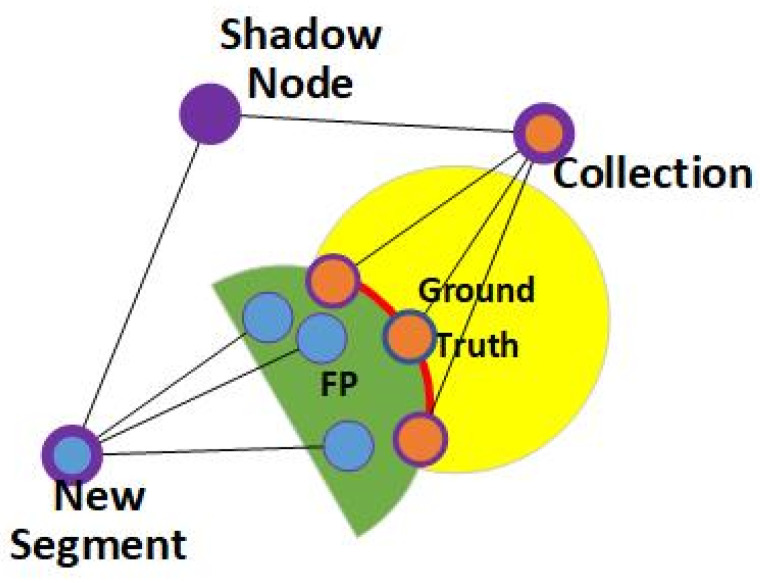
The procedure for building a shadow node 1 using the FP area.

According to the anchored branch, find an inactivated node, $N_{un}$, that is expected to activate adjacent or close to the FP area close to the trigger node. $N_{un}$ should be the highest node of an inactivated branch, such as with node 32 and node 18. The nodes in the same layer as $P_{P1+}$ are chosen with priority. Moreover, their parent nodes, which are marked in red, are recorded as well. There will be two possibilities:

(1) If the node $N_{un}$ is found, then $N_{un}$ will be the trigger node, $T_{p1+}$, of a shadow node 1+. Take the adjacent or nearby FP nodes and their parent nodes to produce a shadow node 1+.
(2) If the node $N_{un}$ is not found, then choose the activated nodes on the boundary between the FP area and the ground truth and make them a collection. After that, choose nodes from the child nodes and make a new segment, $N_{s}$. Produce a shadow node 1- with the collection and trigger node $N_{s}$. Figure 12 briefly describes this process.

As shown in Figure 12, a new collection is built with the nodes activated on the boundary between the ground truth and FP area. Although it is represented by an orange node with a thicker border in Figure 12, the formal node will not be produced. Meanwhile, a new segment will be made to represent the FP area, which is marked in blue with a thicker border. A shadow node 1- will then be produced based on the collection and new segment.

#### 3.3.2. Training Using the FN Area

**Figure 13:**
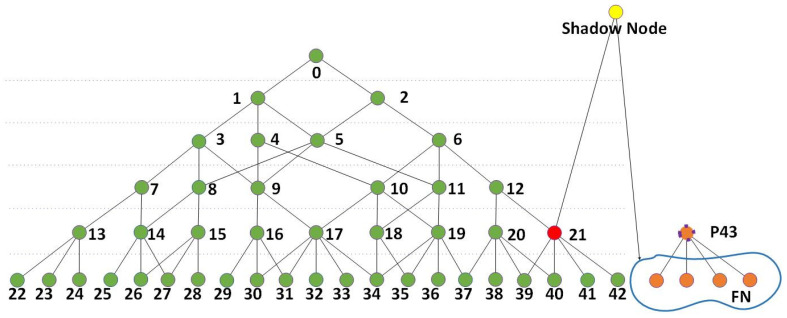
The procedure of adding a shadow node 2. The green nodes are the basic nodes that form the network structure. The orange nodes are child nodes with their unproduced parent nodes (with thicker borders) that form the collection, and the red node is the trigger node. The child nodes that represent the FN areas are marked with a blue circle.

In this case, the purpose of training is to add areas that are not learned or covered by inactivated branches without influencing the activation condition of the nodes in the structure. Therefore, a shadow node 2, which does not change the activation of nodes, is applied to the procedure. The procedure is shown in Figure 13.

Apparently, the FN areas are not covered by activated nodes that contribute to the network structure; thus, a feature node or several feature nodes that represent the FN area are needed. The segment construction is the same as that introduced in [1]. As shown in Figure 13, the orange nodes in FN are produced to cover the area. The nodes in lower layers combine into and form nodes in higher layers until the network structure converges into a single node, which is marked as P43 in Figure 13. It should be noted that P43 is a virtual segment that is only used to obtain its child nodes and is not actually added to the structure. Then, an activated node, $T_{P1\text{-}}$, the area of which is adjacent or close to the FN area, is chosen as the trigger node; the nodes in the same layer as P43 will be chosen with priority, such as with node 21 in Figure 13. Finally, a shadow node 2 is produced with the child nodes in the non-formal segment as the collection and the node $T_{P1\text{-}}$ as the trigger. As a shadow node 2 is computed in the back-tracing procedure after the final node 0 is computed, we put it higher than the layer where node 0 stays.

#### 3.3.3. Training for Distinguishing a Contained Object

In the former design in [1], number “1” was captured from number “7”. This false detection could be alleviated by adding a shadow node 1-CHANGE. The differences between a 1-CHANGE node and a 1- node are the registered layer and if they affect the activation of trigger nodes’ parent nodes.

**Figure 14:**
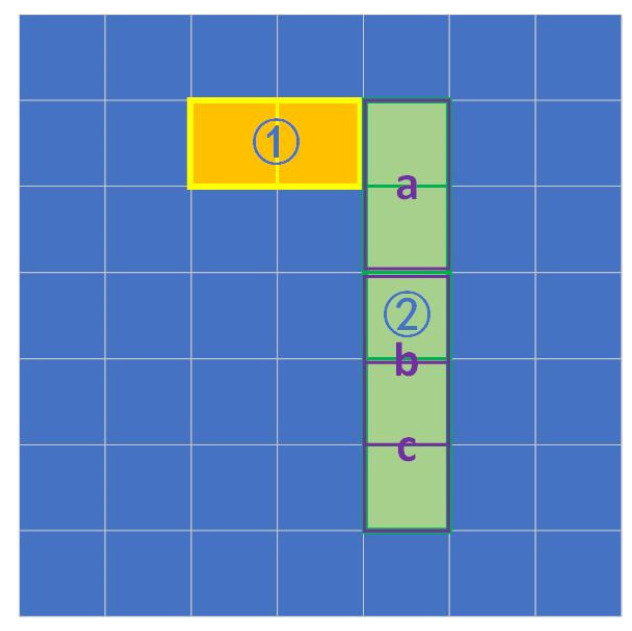
A schematic of the areas involved in node production. The green area numbered ② is the part that incorrectly activates the network representing the number “1”, and the yellow area numbered ① is the area that helps the network to distinguish between “7” and “1”. Areas a, b, and c are three nodes that represent the green area (area b and c cover each other).

The purpose of this rule is to reduce the false activation of nodes that lead to the activation of the structure representing number “1”; therefore, we need to increase the difficulty of activating corresponding nodes for category “1” and keep the nodes for “7” as intact as possible. According to the case of “1” in “7”, the FN part is not included in the network structure for “1”; thus, the FN part will be the condition that makes the final node for “1” inactive when testing on “7”. A schematic of the involved areas can be seen in Figure 14.

Similarly to the construction progress of a shadow node 1-, we build a non-formal segment on the FN area as the collection and choose an activated adjacent node that contributes to the network structure for “1” to be the trigger node. The trigger node’s layer should be one layer higher than the child nodes in the collection. As shown in Figure 14, the area for number “7” consists of areas ① and ②. Apparently, area ② is likely to be treated as number “1”, losing a small part of the border (that between the two areas). Therefore, a promising solution is to control the activation of nodes representing number “1” (which is denoted as node ②) with area ①. Assume that node ② is combined with three nodes, nodes a, b, and c, and node a is adjacent to area ①. Then, a collection is made that represents area ①, and it is used to control the activation of node ②.

It should be noted that in the construction procedure, newly produced segments are used without repeating; thus, the re-edition of the segments in a branch tends to have less influence on other branches of the network structure.

## 4. Results

We tested the performance of the network with or without the training process on the Fashion-Mnist, Omniglot, and Cifar-10 datasets. Moreover, we tested the training for a contained object on part of the original Mnist dataset to compare its performance with the untrained network introduced in [1]. As the training process only runs on activated networks, the training samples were selected to make sure the constructed network was activated. In this section, true positive, true negative, false positive, false negative, and intersection over union are referred to as TP, TN, FP, FN, and IOU, respectively, for short.

Currently, the training method proposed in this paper is the only method that works on correlation-coefficient-based networks, making it hard to conduct a contrast experiment. Additionally, the correlation-coefficient-based network is based on merely two samples (one for construction and another for training), while the existing models take advantage of a larger training dataset, which makes it hard to make a fair comparison.

### 4.1. Training Experiment for Segmentation

#### 4.1.1. Training Using Fashion-Mnist

Fashion-Mnist is a 10-class dataset containing 70,000 28 × 28-sized clothing images. It is more complicated than the Mnist dataset, as the clothing is more variable. As the training progress only runs on activated structures, we set up the experiment as follows:

1. The efficient mask and ground truth of a sample were set according to the pixel values with a threshold of 0.03.
2. Ten pictures (one picture per category) were chosen as the construction dataset to construct the network structure for each category.
3. The constructed network was tested on the database. The testing samples were randomly selected and denoted as $S_{test}$. As the training process does not change the target’s recognition rate, the unidentified samples were ignored.
4. Another 10 pictures (one picture per category) were chosen for which the target could be captured as the training dataset to train the structure for each category.
5. The trained network was trained on the selected database $S_{test}$. The improvements in IOU are shown in Table 1.

It can be seen that, after training, the IOU of the segmented target increased by 4.83%. More specifically, after the training on FP areas alone, the IOU increased by 1.85% (Table 2), and after the training on FN areas alone, the IOU increased by 2.37% (Table 3).

As the IOU increase may also involve changes in the union area and may also introduce new FP and FN pixels, the IOU increase that comes from FP and FN areas altogether does not equal the sum of IOU increases that come from FP areas and FN areas, respectively.

**Figure 15:**
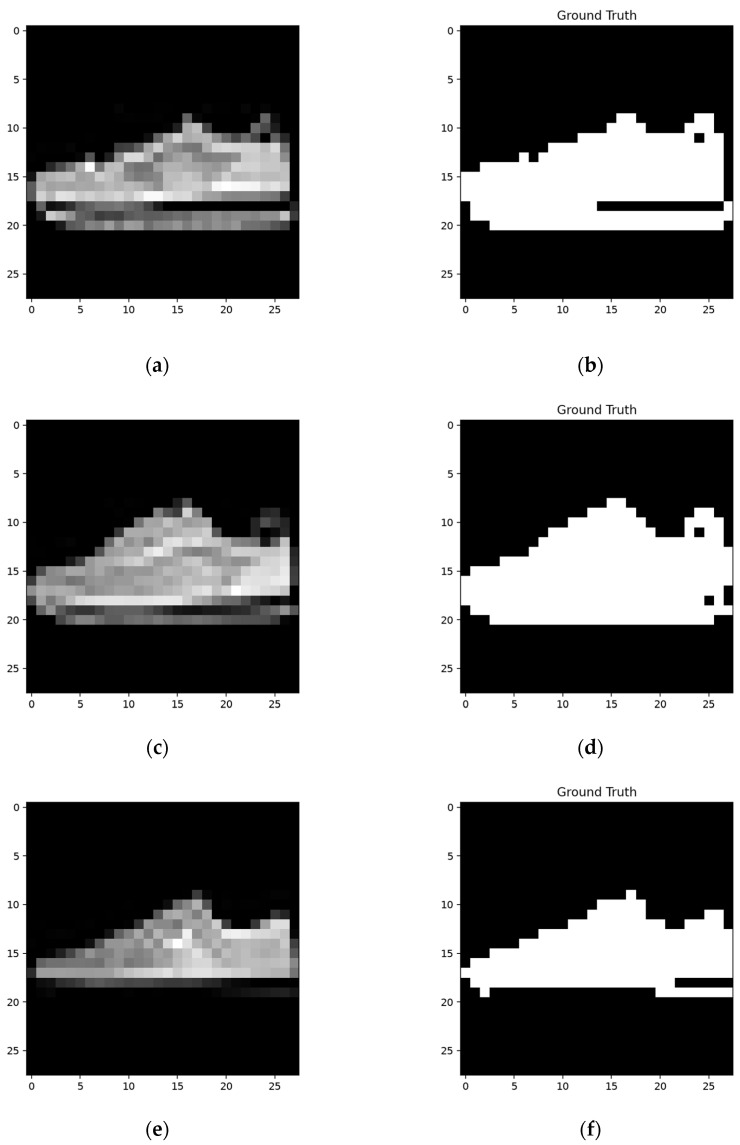
One set of the constructing, training, and testing samples. (**a**,**c**,**e**) are original grayscale pictures, and (**b**,**d**,**f**) are the corresponding ground truths.

We can see the training details in the following network; Figure 15 shows a set of constructing, training, and testing samples regarding the network structure.

**Figure 16:**
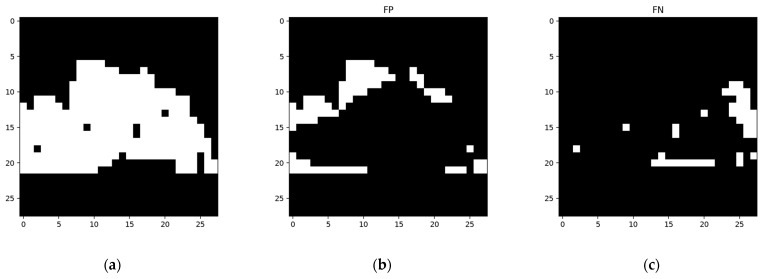
Detection results for the training sample of the network structure built using the samples in Figure 15: (**a**) segmentation result using the training sample; (**b**) FP area using the training sample; (**c**) FN area using the training sample.

The network was constructed on the constructing sample (Figure 15a) and then worked on the training sample (Figure 15c). Then, the network was trained according to its result when using the training sample. The segmentation results using the training sample, along with the corresponding FP and FN, areas are shown in Figure 16.

**Figure 17:**
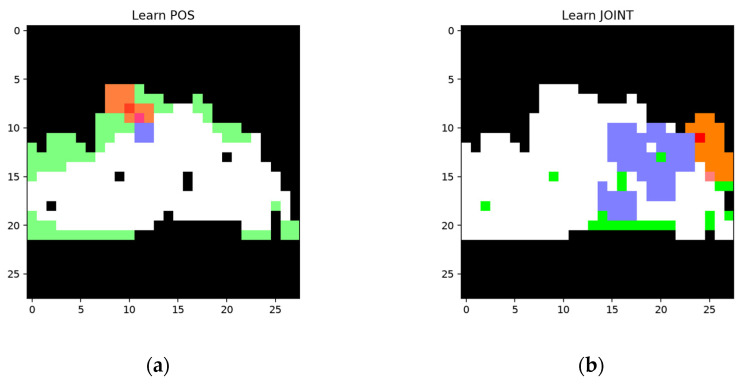
One pair of features that learned using the FP and FN areas, respectively (based on the same network structure as in Figure 16): (**a**) one of the features that learned using the FP areas, where the segmented areas are marked in white, the FP areas are marked in green, the area covered by a trigger node is marked in blue, and the area covered by the collection is marked in red; (**b**) one of the features learned using the FN areas, where the segmented areas are marked in white, the FN areas are marked in green, the area covered by the trigger node is marked in blue, and the area covered by the collection is marked in red.

The learned features when using the training sample are based on the FP and FN areas. One of the features learned using the FP and FN areas, respectively, are shown in Figure 17.

Apparently, the shadow node in Figure 17a aims to inactivate the parts of the feature nodes that are not supposed to be activated in the FP area, while the shadow node in (b) aims to add areas that are supposed to be activated. 

**Figure 18:**
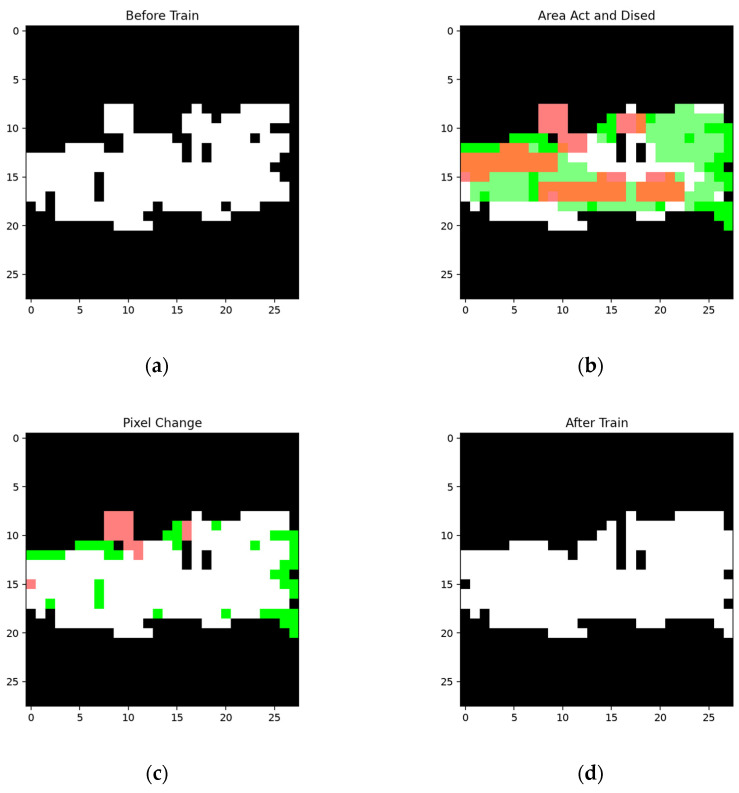
Segmentation results and pixel changes for the testing sample using the same network structures as in Figure 16 and Figure 17: (**a**) segmentation result before training; (**b**) area covered by activated shadow nodes, where the white areas represent the segmentation result before training and the areas covered by the shadow nodes that are supposed to be added and inactivated are marked in green and red, respectively; (**c**) pixel change for the segmented area after training, where the added pixels and inactivated pixels are marked in green and red, respectively; (**d**) segmentation result for the testing sample after training.

The collection of the shadow node in (a) is produced to cover the FP areas and decreases the unwanted area by around 14 pixels. If the trigger node and the collection of the shadow node in (b) are activated, the FN area will decrease by around 22 pixels. The segmentation results using the testing sample and the pixel comparison are shown in Figure 18.

It should be noted that although the inactivated features cover part of the segmentation areas, the redundant nodes (which are produced in the network construction phase when requiring the nodes to cover each other) keep the more reliable areas (which are covered by more activated nodes) unchanged. Such a structure keeps the result more stable. The statistical results of pixels before and after training are shown in Table 4.

**Figure 19:**
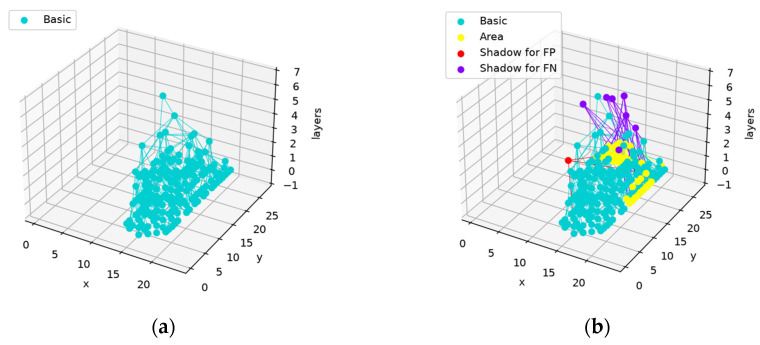
Network structure before and after training (based on the same network structure as in Figure 16, Figure 17 and Figure 18): (**a**) network structure before training; (**b**) network structure after training. The basic nodes produced in the construction phase forming the main structure are marked in blue, the basic nodes that are produced to represent the FN areas are marked in yellow, the shadow nodes trained on the FP areas are marked in red, and the shadow nodes trained on the FN areas are marked in purple.

The network structure before and after training is shown in Figure 19.

The training process does not change the main structure and serves as a minor adjustment to the features that are needed and captured. This design keeps the main structure stable.

**Figure 20:**
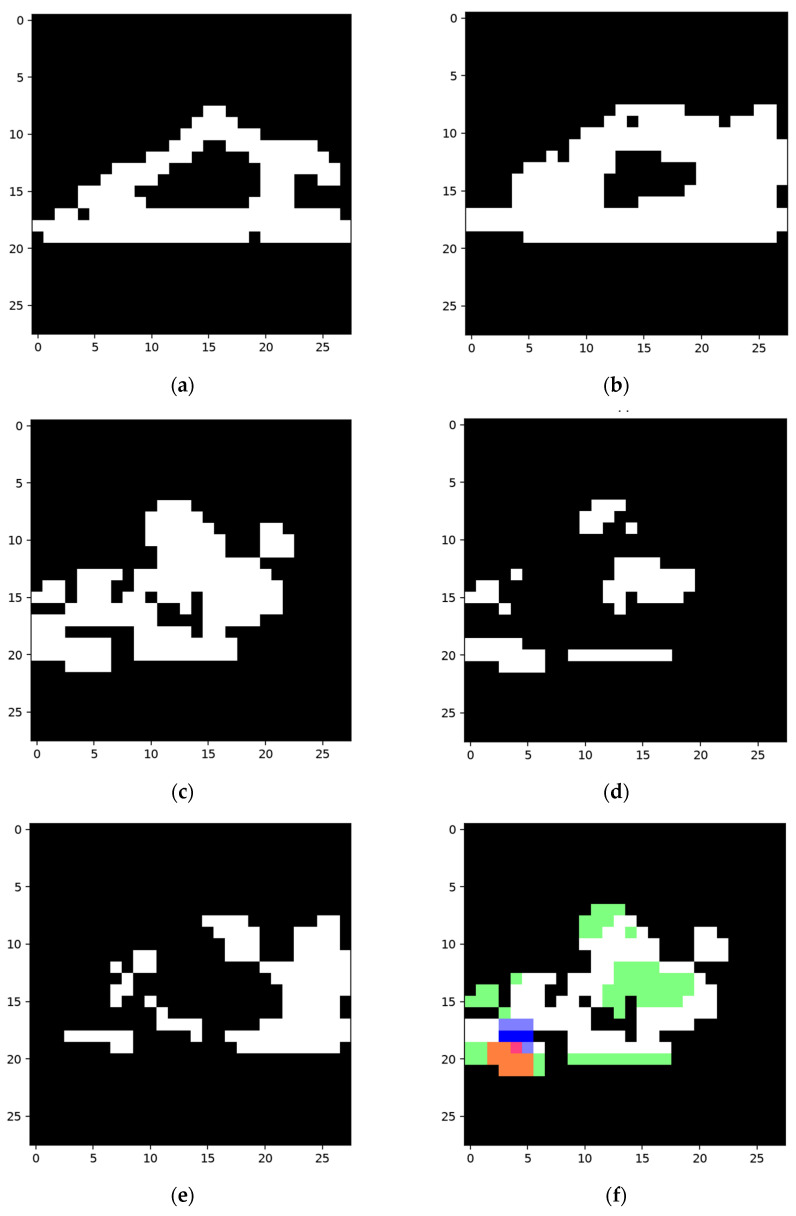

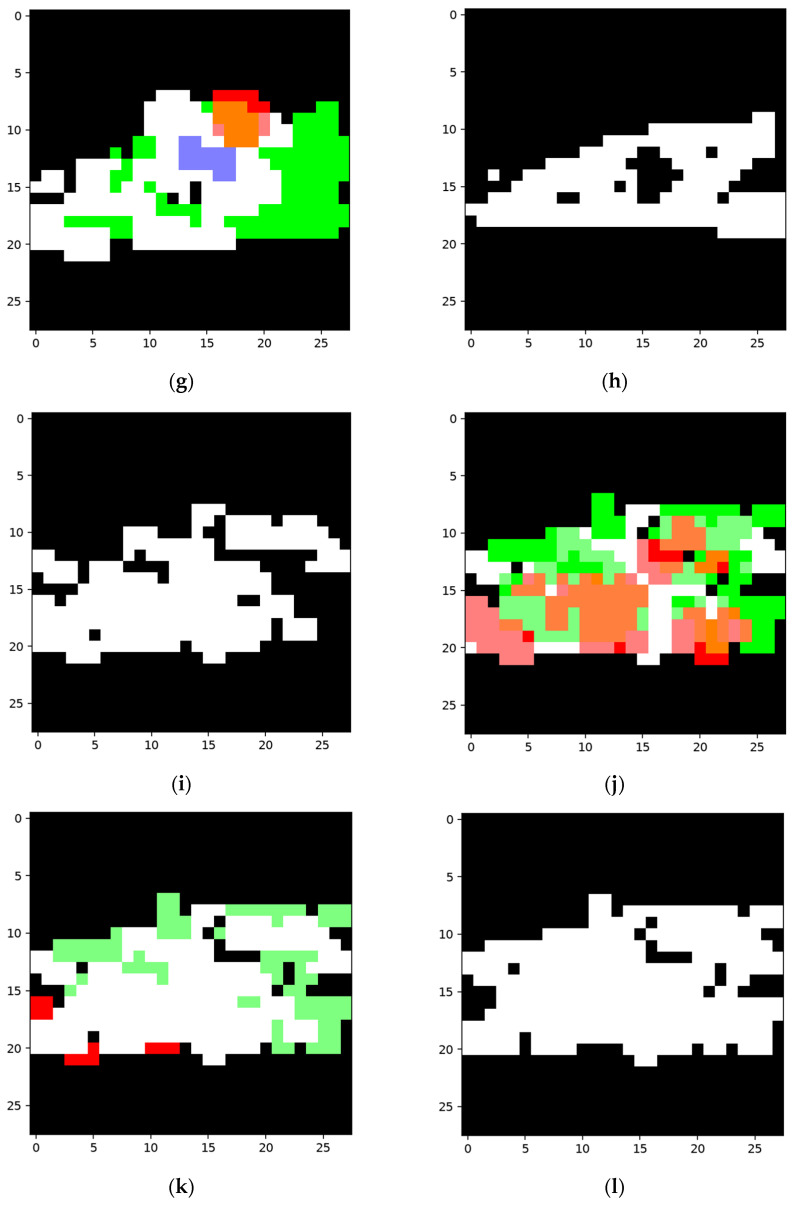

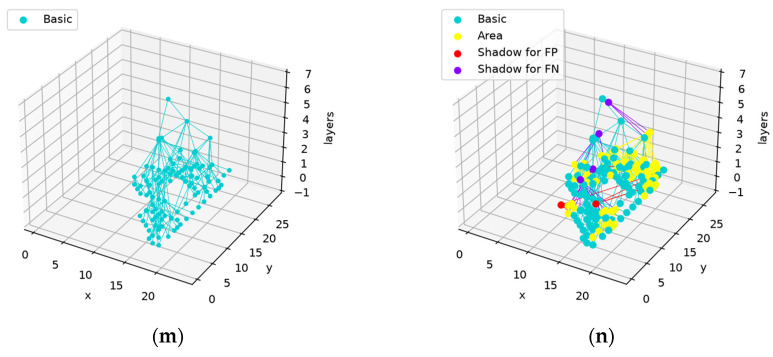
Another set of networks and their results for a different category using the Fashion-Mnist dataset: (**a**) efficient map of the constructing sample; (**b**) efficient mask of the training sample; (**c**) segmentation result for the training sample; (**d**) FP area; (**e**) FN area; (**f**) one of the shadow node 1+s produced based on the FP area, the trigger of which is marked in blue and the collection is marked in red, with the FP area marked in green; (**g**) one of the shadow node 2s produced based on the FN area, the trigger of which is marked in blue and the collection is marked in red, with the FN area marked in green; (**h**) efficient map of the testing sample; (**i**) segmentation result for the testing sample before training; (**j**) the area added by the activated shadow node 2s (marked in green) and the area disabled by activated shadow node 1+s (marked in red); (**k**) pixel change for the segmentation result, where the added pixels are marked in green and the deleted pixels are marked in red; (**l**) segmentation result for the testing sample after training; (**m**) network structure before training; (**n**) network structure after training.

Figure 20 shows another set of network and segmentation results. Their pixel statistics are shown in Table 5.

The IOU improvement after training may differ due to the randomness of the feature nodes produced or chosen to form shadow nodes. As shown in Table 4 and Table 5, if the shadow nodes introduce more wrongly classified pixels into the segmentation result, they may only increase the IOU slightly or, in some cases, downgrade the performance of the network. However, if they are chosen properly, they could improve the network performance via merely one picture. The distribution of the nodes in a network structure indicates that the nodes are produced based on the location and complexity of the target, which leads to a primitive attention mechanism.

It should be taken into consideration that the network is constructed and trained using merely one sample, respectively, without any pre-training. As far as we know, the model we proposed in [1] with the training strategy proposed in this paper is the only model that can accomplish such few-shot detection and segmentation without pre-training.

#### 4.1.2. Training Using Omniglot

Omniglot is a database covering 1623 handwritten characters from 50 different languages, with 20 samples for each character. When compared to the Mnist and Fashion-Mnist datasets, the samples in Omniglot only contain pixel values of 0 and 1. We decreased the 2 × 2 sampling to make more complicated borders and pixels as well as accelerate the calculation. The samples were set as follows:

1. The efficient mask and ground truth of a sample were set according to the pixel values with a threshold of 0.97, as the black parts stand for the foreground, which is contrary to the samples in Mnist and Fashion-Mnist.
2. A character is randomly chosen, and one of the 20 samples was chosen as the construction sample to construct the network structure.
3. The constructed network was tested on the samples of the same character. The testing samples were randomly selected and denoted as $S_{test}$. As the training process does not change the recognition rate of the target, the unidentified samples were ignored.
4. Another sample of the same character was chosen for which the target could be captured and the network activated as the training sample to train the structure.
5. The trained networks were trained on the selected dataset $S_{test}$.
6. The procedures were repeated. The average improvement in IOU coming from the chosen samples reached 4.02%.

**Figure 21:**
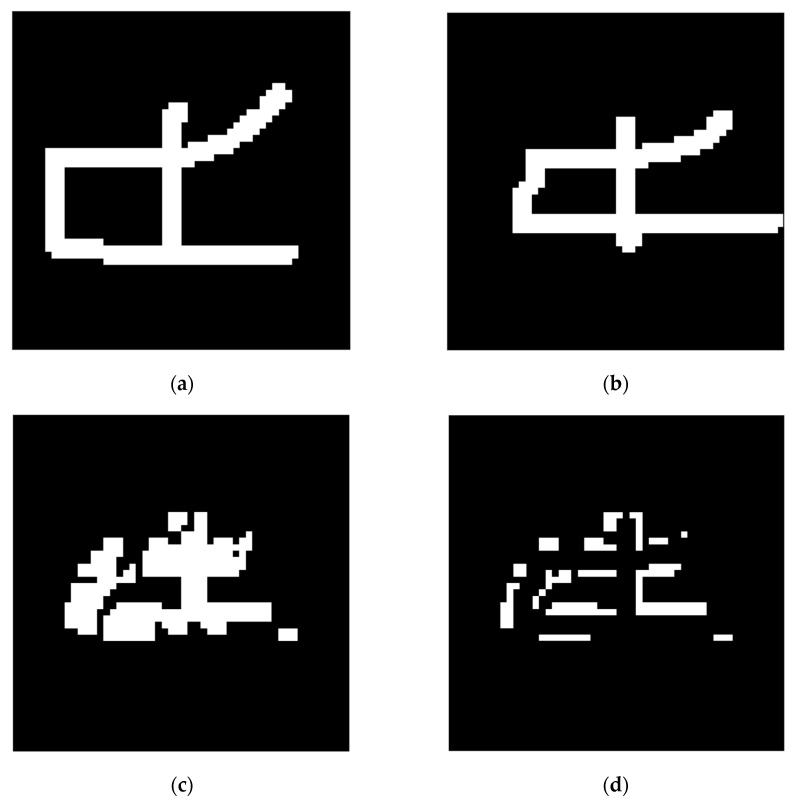

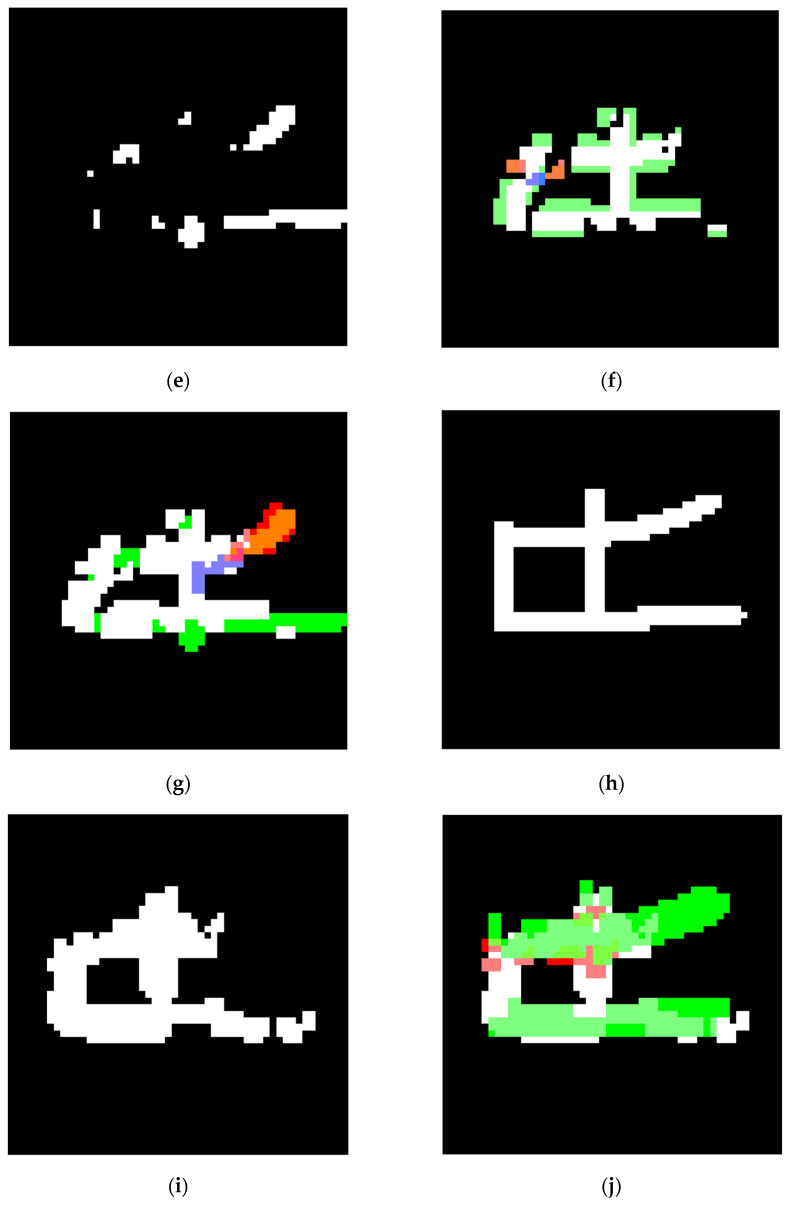

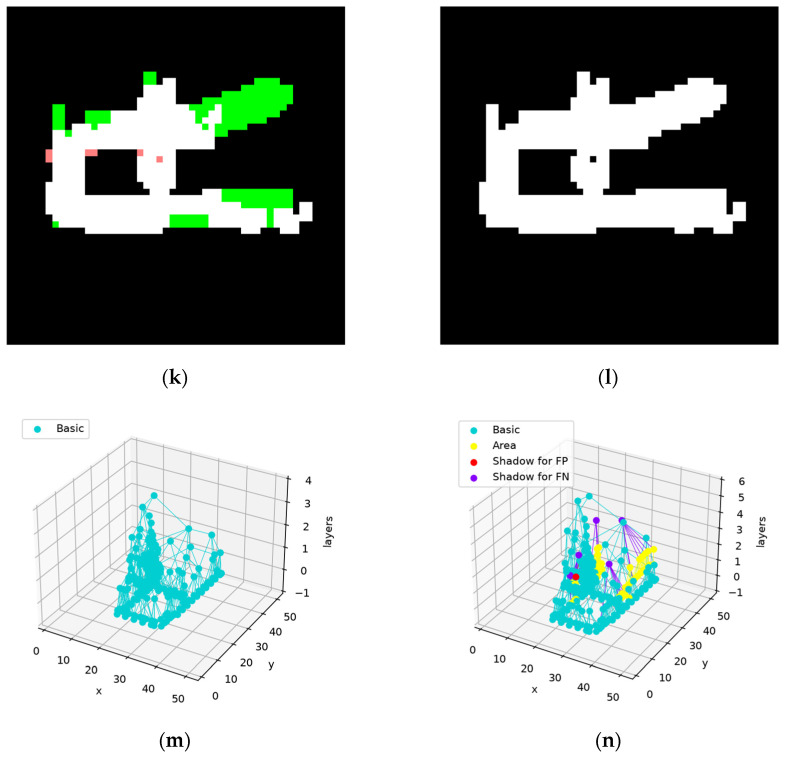
A set of networks and their results using the Omniglot dataset: (**a**) efficient map of the constructing sample; (**b**) efficient mask of the training sample; (**c**) segmentation result for the training sample; (**d**) FP area; (**e**) FN area; (**f**) one of the shadow node 1+s produced based on the FP area, the trigger of which is marked in blue and the collection is marked in red, with the FP area marked in green; (**g**) one of the shadow node 2s produced based on the FN area, the trigger of which is marked in blue and the collection is marked in red, with the FN area marked in green; (**h**) efficient map of the testing sample; (**i**) segmentation result for the testing sample before training; (**j**) the area added by activated shadow node 2s (marked in green) and the area disabled by activated shadow node 1+s (marked in red); (**k**) pixel change for the segmentation result, where the added pixels are marked in green and the deleted pixels are marked in red; (**l**) the segmentation result for the testing sample after training; (**m**) network structure before training; (**n**) network structure after training.

**Figure 22:**
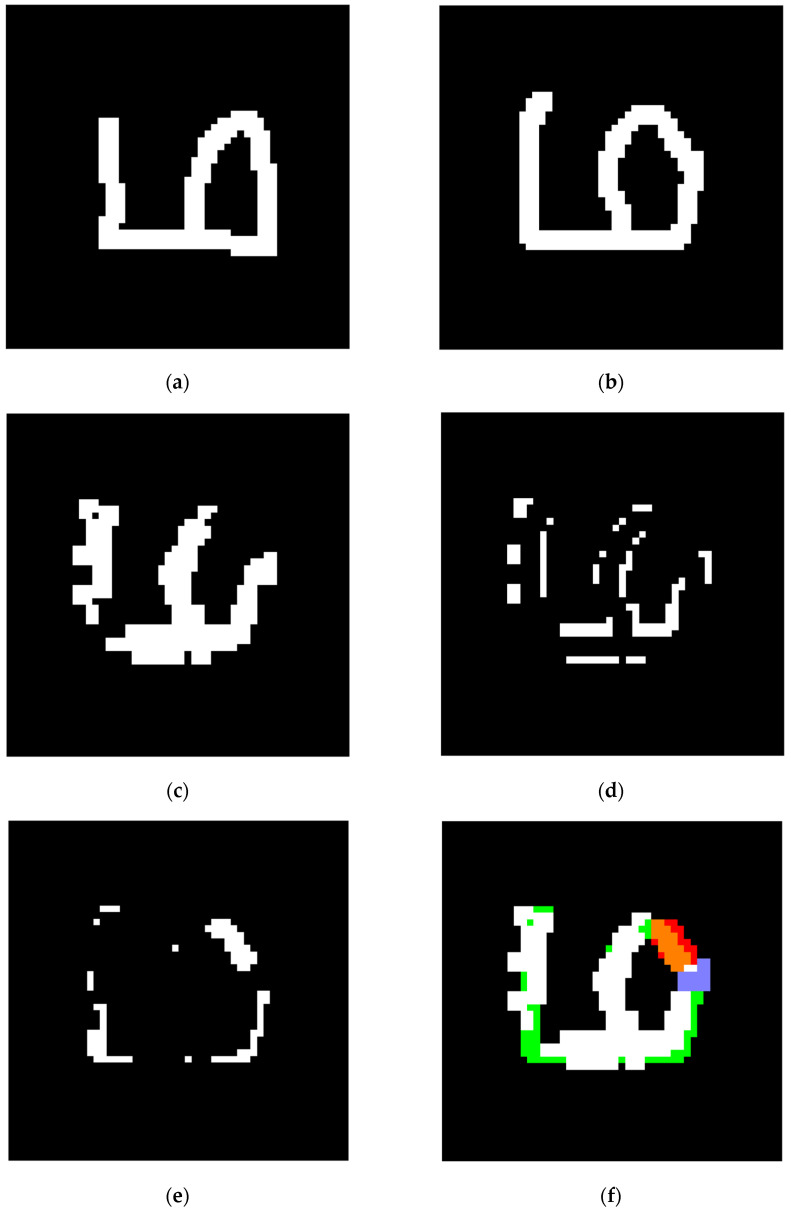

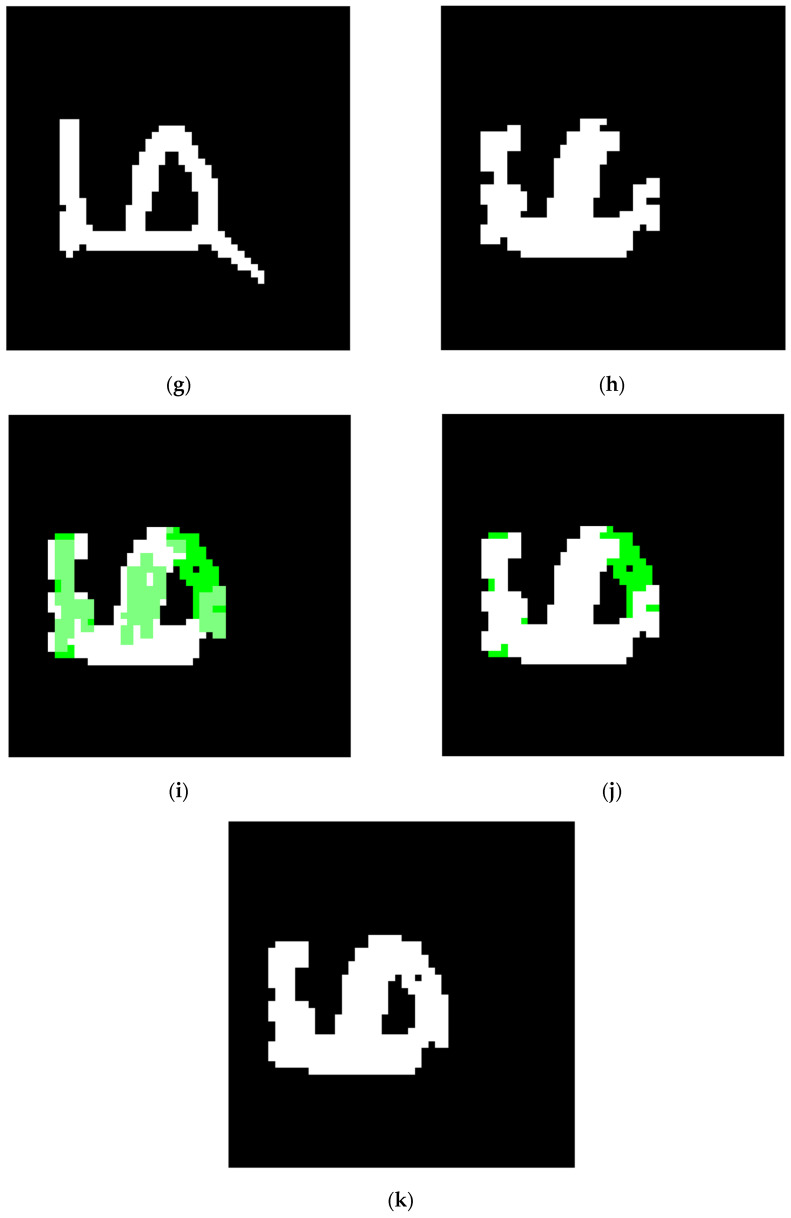

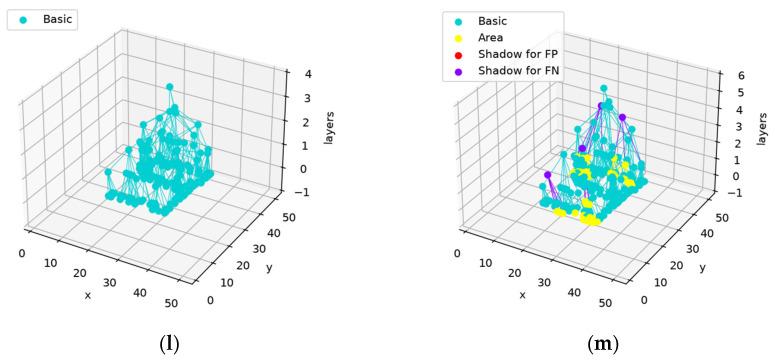
Another set of networks and their results using a different category in the Omniglot dataset: (**a**) efficient map of the constructing sample; (**b**) efficient mask of the training sample; (**c**) segmentation result for the training sample; (**d**) FP area; (**e**) FN area; (**f**) one of the shadow node 2s produced based on the FN area, the trigger of which is marked in blue and the collection is marked in red, with the FN area marked in green; (**g**) efficient map of the testing sample; (**h**) segmentation result for the testing sample before training; (**i**) the area added by activated shadow node 2s (marked in green) and the area disabled by activated shadow node 1+s (marked in red); (**j**) pixel change for the segmentation result, where the added pixels are marked in green and the deleted pixels are marked in red; (**k**) segmentation result for the testing sample after training; (**l**) network structure before training; (**m**) network structure after training.

Figure 21 and Figure 22 show two sets of the tested samples, and their statistical results of pixels before and after training are shown in Table 6 and Table 7.

As the FP areas using Omniglot tend to be narrow and the activated kernels rarely fall completely within the areas, there are a few shadow nodes learned from the FP areas. However, the shadow node 2s produced from the FN areas can supplement missing parts efficiently if the training samples happen to be reliable and present features that resemble the testing samples, as is also shown in Figure 22.

#### 4.1.3. Training Using Cifar-10

**Figure 23:**
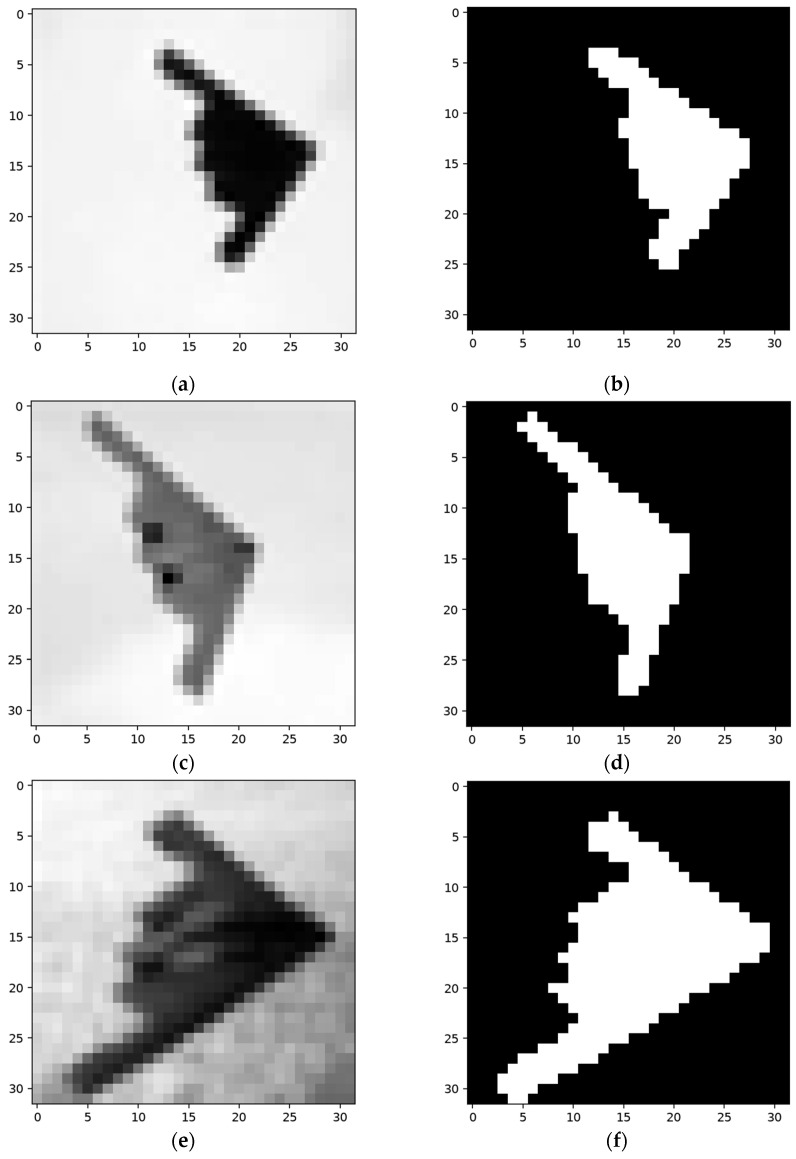
One set of the constructing, training, and testing samples in the Cifar-10 dataset. (**a**,**c,e**) are grayscale pictures converted from color versions, and (**b**,**d**,**f**) are the corresponding ground truths.

Cifar-10 contains 10 categories of color images. As the targets in the samples can vary greatly in angle, texture, and color, and the features obtained from a single sample are too limited for all the situations, the images were selected to ensure that the network activates using the training and testing samples. Due to the complexity of the samples, efficient masks are either drawn by hand or re-edited after being segmented based on a chosen threshold. Moreover, the RGB pictures are converted into grayscale pictures. The testing and training procedures resemble those in the experiments in Section 4.1.1 and Section 4.1.2. Figure 23 shows one set of construction, training, and testing samples.

**Figure 24:**
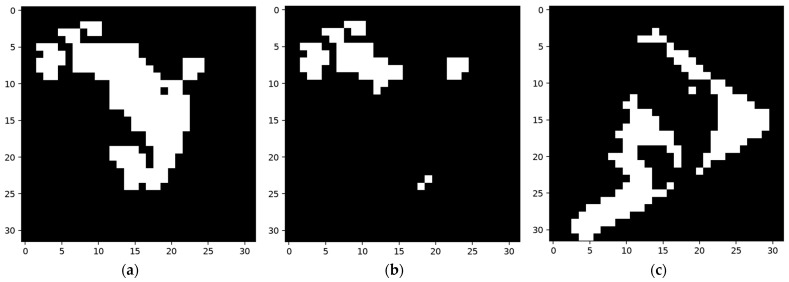
Detection results for the training sample of a network structure built using the samples in Figure 23: (**a**) segmentation result for the training sample; (**b**) the FP area for the training sample; (**c**) the FN area for the training sample.

The segmentation results using the training sample and the corresponding FP and FN areas are shown in Figure 24.

**Figure 25:**
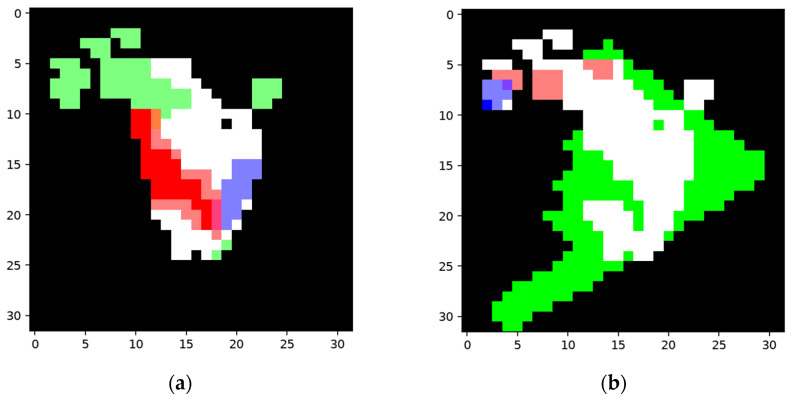
One pair of features learned using the FP and FN areas, respectively (based on the same network structure as in Figure 24): (**a**) one of the features learned using the FP areas, where the segmented areas are marked in white, the FP areas are marked in green, the area covered by trigger node is marked in blue, and the area covered by the collection is marked in red; (**b**) one of the features learned using the FN areas, where the segmented areas are marked in white, the FN areas are marked in green, the area covered by trigger node is marked in blue, and the area covered by the collection is marked in red.

One pair of the shadow nodes produced, respectively, using the FP and FN areas is shown in Figure 25.

**Figure 26:**
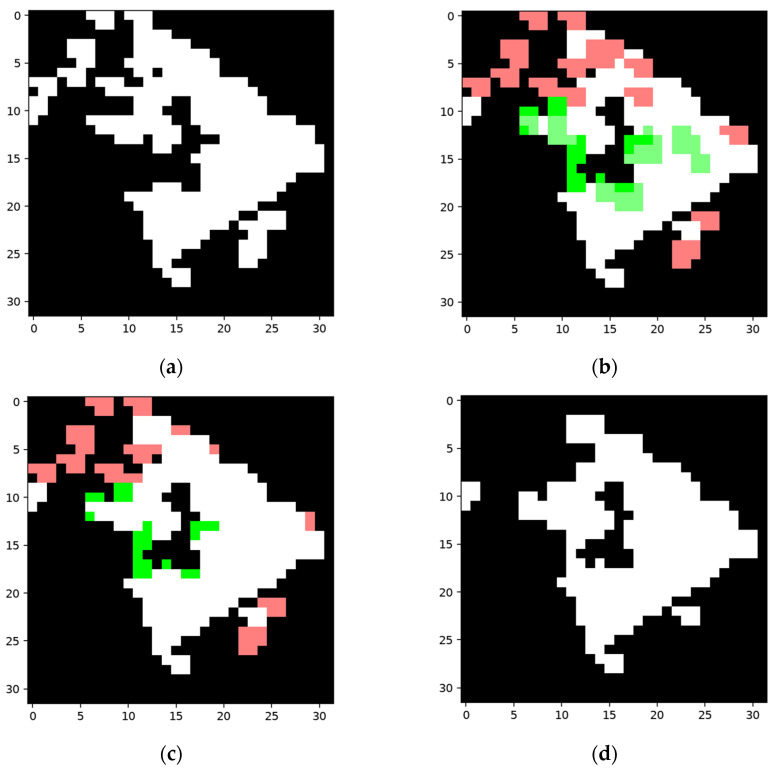
Segmentation results before and after training (based on the same network structure in Figure 24 and Figure 25): (**a**) segmentation result for the testing sample before training; (**b**) the area added by activated shadow node 2s (marked in green) and the area disabled by activated shadow node 1+s (marked in red); (**c**) pixel change for the segmentation result, where the added pixels are marked in green and the deleted pixels are marked in red; (**d**) segmentation result for the testing sample after training.

After the shadow nodes are produced, the trained network was tested on the testing sample again. The segmentation results before and after training, as well as the pixel changes, are shown in Figure 26.

**Figure 27:**
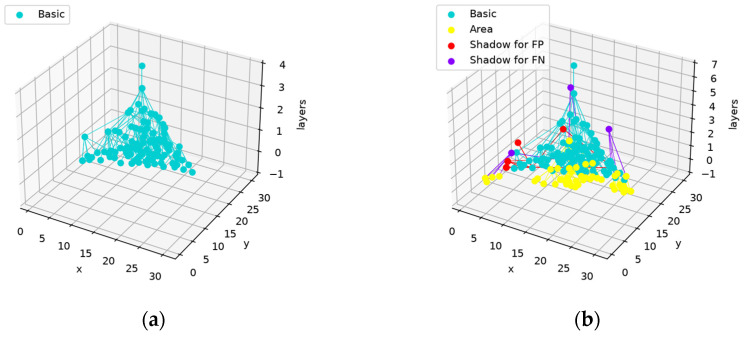
Network structures before and after training (based on the same network structure as in Figure 24, Figure 25 and Figure 26): (**a**) network structure before training; (**b**) network structure after training. The basic nodes produced in the construction phase that form the main structure are marked in blue; the basic nodes that were produced to represent the FN areas are marked in yellow; the shadow nodes trained using the FP areas are marked in red; the shadow nodes trained using the FN areas are marked in purple.

The network structures before and after training are shown in Figure 27.

The statistical results of pixels before and after training are shown in Table 8.

**Figure 28:**
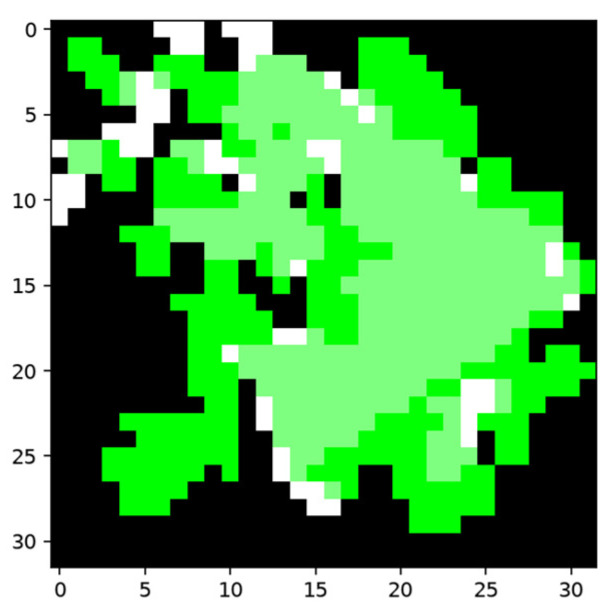
Undesirable result because of a bad combination of features and inaccurate anchoring. The segmentation result before training is marked in white, and the area covered by activated shadow node 2s is marked in green. The white and green areas cover each other, and the overlapped area is in a lighter shade of green.

The average IOU increase using Cifar-10 is 3.89%. The effectiveness of the area deleted and added to the segmentation results depends on the accuracy of the anchored and activated feature nodes. As randomness lies in the selection of features when producing the neuron nodes, there could be bad combinations of nodes that activate the shadow nodes using unwanted locations; this leads to destructive results, as shown in Figure 28.

### 4.2. The Experiments Using Training to Distinguish Contained Samples

The purpose of the experiment is to prove the effectiveness of the training rule that distinguishes the contained object from the larger one. As this problem was discovered in the earlier version proposed in [1], we continued using the Mnist dataset to show the improvement. The testing samples were selected to ensure that there were false detection problems, and after training, the networks were tested using the same set of samples to compare the results.

**Figure 29:**
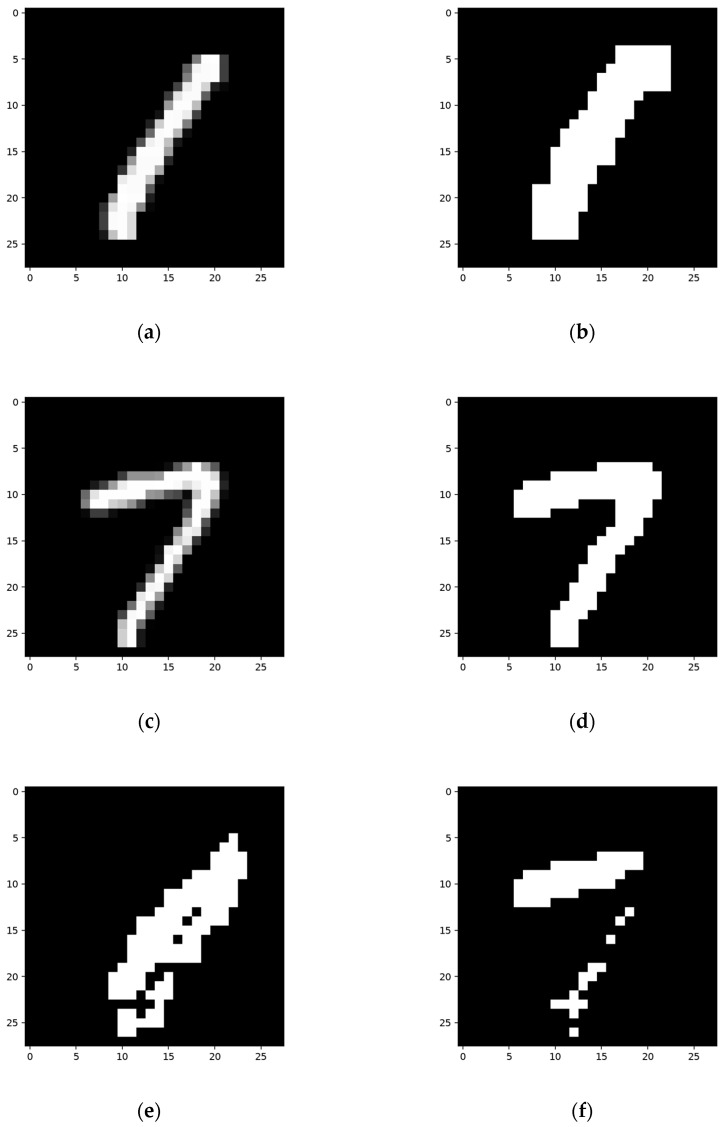
Constructing sample and training sample. (**a**) Constructing sample for network detecting category “1”; (**b**) the efficient map of the constructing sample; (**c**) training sample in category “7”, which is wrongly classified as “1”; (**d**) efficient map of the sample in (**c**); (**e**) segmentation result of the network using the sample in (**c**); (**f**) the areas that were not detected, taken as the difference between two categories.

As shown in Figure 29, a sample of category ”1” and its corresponding efficient mask were used to construct the network, and a misclassified sample of category “7” was used to train the network to distinguish the two categories.

**Figure 30:**
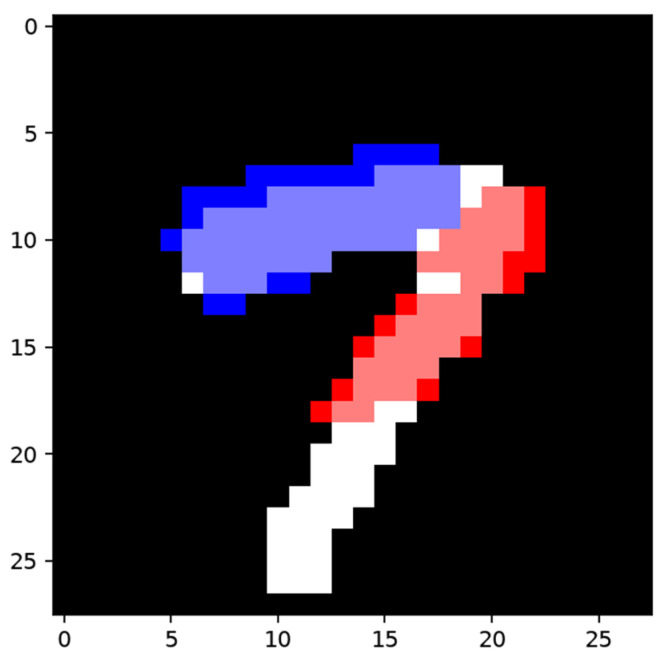
Produced shadow node with trigger and collection. The efficient area of the training sample is marked in white, the area covered by the collection is marked in blue, and the area covered by the trigger node is marked in red.

The collection of the shadow node-CHANGE was then built according to Figure 29f. The built collection based on Figure 29f and its trigger node chosen from existing nodes are shown in Figure 30.

The area in blue is the area covered by nodes built using the surplus area, forming the collection part of a shadow node 1-CHANGE. The red part was chosen from activated adjacent features in the same layer from the main network structure. The activation of the blue part will inactivate the feature node for which the area is marked in red.

**Figure 31:**
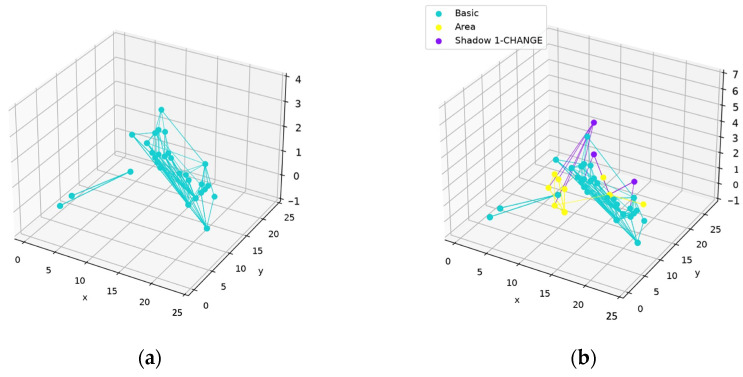
Network structures before and after training: (**a**) network structure before training; (**b**) network structure after training.

The network structures before and after training are shown in Figure 31.

**Figure 32:**
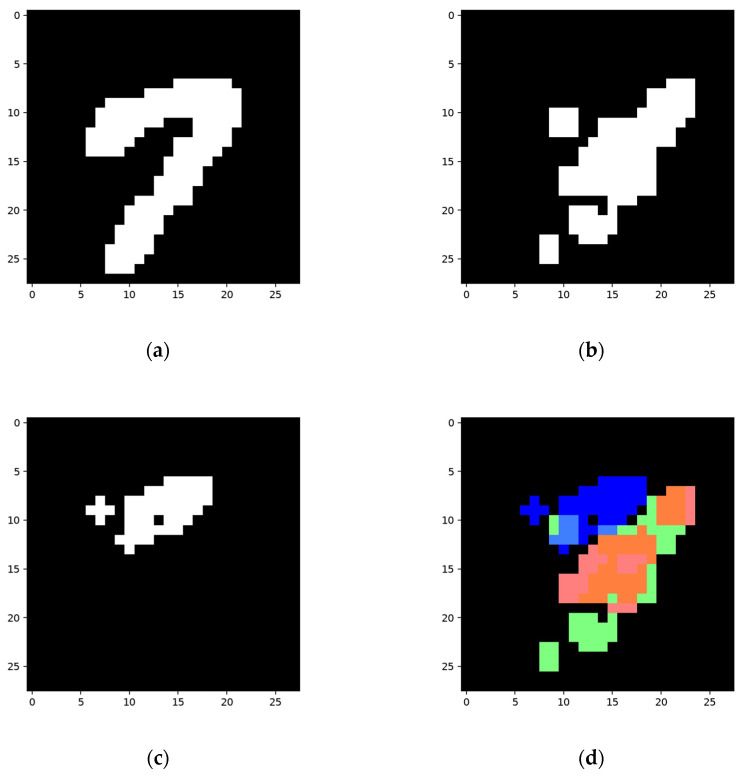
Activated feature nodes while checking sample “7”. (**a**) shows the ground truth of the testing sample; (**b**) is the wrongly recognized and segmented area by the untrained network; (**c**) shows the feature that prevents the activation of the network; (**d**) shows how the network is inactivated. The area covered by activated nodes in the collection of a shadow node 1-CHANGE is marked in blue, and the red area marks the inactivated trigger node. The area covered by unchanged nodes is marked in green. The areas cover each other.

The yellow nodes in Figure 31b are produced to represent the area used to distinguish two categories. As with the network shown in Figure 19 and Figure 20m,n, the newly produced nodes do not affect the main structure. However, as the activated nodes in the feature map are inactivated before the parent nodes are registered as being activated, their absence will lead to the inactivation of the whole network structure that wrongly classifies “7” as “1”. The results are shown in Figure 32.

As shown in Figure 32, the area in (c) (which is marked in blue in (d)) is the feature for the collection of a shadow node 1-CHANGE to be activated. The red area in (d) is the area covered by the activated trigger node that contributes to the activation of the structure for number “1”. The activation of the collection inactivates the trigger, therefore inactivating the final node of the network and thus correcting the classification results. The green area is the area covered by the final node’s activated child nodes, which remain unchanged. The segmentation result of the trained network is not displayed, as the network will not be activated after training.

**Figure 33:**
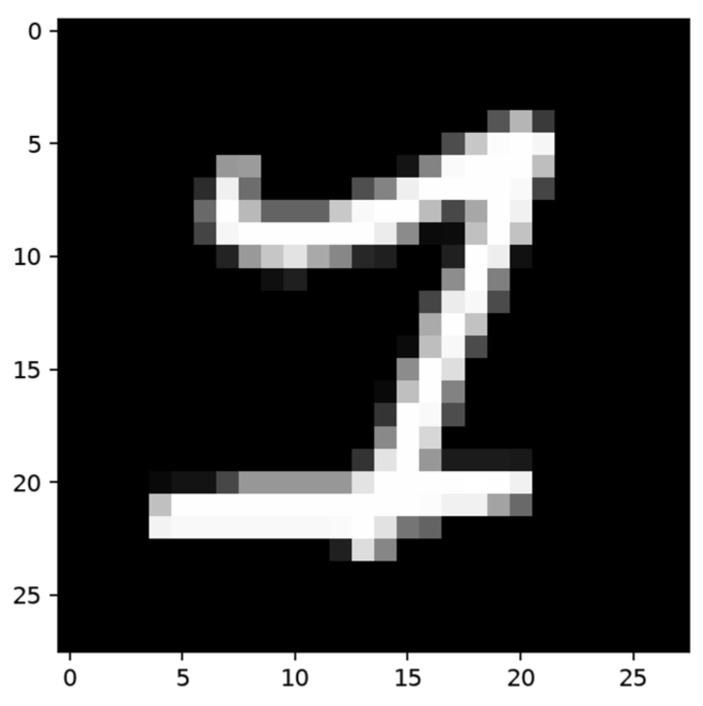
One of the samples that failed to be correctly classified as “1” after training.

This training process reduces 27.32% of the misclassification from “7” to “1”. Meanwhile, as some of the similar features in part of the samples in category “1” are also detected, as is shown in Figure 33, the recall rate of category “1” dropped by 2.86%. 

## 5. Discussion

The experiment results show that this training strategy helps the correlation-coefficient-based network develop its segmentation performance. Shadow nodes 1+, 1-, and 2 change the feature maps that record activated nodes rather than changing the main network structure. This procedure imitates the temporary correction of misjudgment regarding the use of unfamiliar patterns when humans are trying to figure out the difference between a new object and a learned one. The temporary correction will not affect the original network structure or the activation of higher nodes. Consequently, such editing using the feature map will not influence the classification result, which reduces the chance of deterioration resulting from inappropriate features from stochastic samples. When it comes to wrongly classified samples that are contained in other samples, where classification results should be corrected, a shadow node 1-CHANGE will be introduced to adjust the activation of the upper branch.

The logic involved in the network structure, such as XOR, could be added on-demand as long as the corresponding nodes are able to be anchored. This design makes the network structure more flexible when carrying out high-level feature recognition.

## 6. Conclusions

In this paper, we proposed a structure-based training strategy for a correlation-coefficient-based network and three kinds of shadow nodes that supplement the function and performance of the network. As the correlation-coefficient-based network is an OSD network built using a single sample without pre-training, its performance is restricted by the limited samples. Therefore, we introduced the training strategy along with more shadow nodes to address the knowledge accumulation problem. Compared to existing training methods, the strategy proposed in this paper focuses on the network structure rather than the parameters of the neurons and connections. The strategy readjusts the activation state of nodes and branches based on FP and FN pixels rather than the value of the error. In the experiment, we proved that such a strategy improves the IOU of the segmentation result by 4.83% when using the Fashion-Mnist dataset, 4.02% when using the Omniglot dataset, and 3.89% when using the Cifat-10 dataset. Moreover, the classification error between categories for which the areas are contained within another decreased by 27.32%. This strategy, along with the shadow nodes, enables the network to improve while more reliable samples accumulate during the process, thus making the network more practical, especially in simple target detection projects that start with few training examples. This strategy is also a supplement to the non-gradient-based parameter-gaining strategy. As far as we know, the correlation-coefficient-based network supplemented with this strategy and shadow nodes is the only model that can be constructed and trained using a single sample without pre-training. This is also a new attempt at the exploration of imitating humans to learn using a low number of references.

In the design period, we planned to allow for the shadow nodes to change the combination of an ordinary segment according to the error map. However, the randomness of the sample may downgrade the performance of the network when detecting learned patterns. Moreover, as the FP and FN areas require back-tracing results, the final node must be activated. This limits the application of the training strategy. In future work, we will address simplifying the calculation and improving the network’s ability to decide which part should be edited. This also requires development in biological fields. It could be inferred from the calculation process that computing a single neuron is more complicated than computing in convolutional neural networks. However, when the target is relatively small with fewer features, it will reach a balance between computing cost and training consumption. As infant models, the networks proposed in [1] and in this paper face challenges due to their immaturity. With a more reliable anchoring process and better computational optimization in future work, this model could serve as a lightweight detection network in projects lacking training samples or demanding fast launching. Additionally, although such a training strategy cannot be applied to existing NN models directly, there is a possibility that it may be combined with existing training strategies. We will leave this part to future work. The main codes of the functions mentioned in this paper are available at https://github.com/J-Su-code/CorCoe-Main-Funcs (accessed on 28 September 2024).

## Figures and Tables

**Table 1:** The improvements in the IOU of the networks before and after training.

Category	0	1	2	3	4	5	6	7	8	9
Before Training	52.43%	54.17%	54.24%	51.08%	53.25%	43.87%	42.92%	55.28%	54.64%	50.61%
After Training	57.14%	58.86%	59.17%	55.91%	57.93%	48.35%	47.76%	60.33%	59.79%	55.57%

The samples were selected to ensure that the networks were activated on both the training and testing samples.

**Table 2:** The improvements in the IOU of the networks before and after training (learned using the FP area only).

Category	0	1	2	3	4	5	6	7	8	9
Before Training	52.43%	54.17%	54.24%	51.08%	53.25%	43.87%	42.92%	55.28%	54.64%	50.61%
After Training	54.35%	56.04%	56.29%	52.87%	54.97%	45.59%	44.82%	57.16%	56.37%	52.55%

The samples were selected to ensure that the networks were activated on both the training and testing samples.

**Table 3:** The improvements in the IOU of the networks before and after training (learned using the FN area only).

Category	0	1	2	3	4	5	6	7	8	9
Before Training	52.43%	54.17%	54.24%	51.08%	53.25%	43.87%	42.92%	55.28%	54.64%	50.61%
After Training	54.71%	56.65%	56.72%	53.55%	55.81%	46.04%	45.27%	57.64%	56.90%	52.85%

The samples were selected to ensure that the networks were activated on both the training and testing samples.

**Table 4:** Pixel statistics before and after training.

	Ground Truth		Detected		TP	TN	FP	FN	Union	IOU
	P	N	P	N						
Before Training	180	604	228	556	148	524	80	32	260	56.92%
Train On FP Only	180	604	204	580	141	541	63	39	243	58.02%
Train On FN Only	180	604	269	515	166	501	103	14	283	58.66%
After Training	180	604	254	530	164	514	90	16	270	60.74%

P: positive; N: negative; TP: true positive; TN: true negative; FP: false positive; FN: false negative; IOU: intersection over union.

**Table 5:** Pixel statistics before and after the training of the network and the samples in Figure 20.

	Ground Truth		Detected		TP	TN	FP	FN	Union	IOU
	P	N	P	N						
Before Training	169	615	223	561	123	515	100	46	269	45.72%
Train On FP Only	169	615	204	580	120	531	84	49	253	47.43%
Train On FN Only	169	615	305	479	168	468	147	11	361	50.00%
After Training	169	615	294	490	156	477	138	13	307	50.81%

P: positive; N: negative; TP: true positive; TN: true negative; FP: false positive; FN: false negative; IOU: intersection over union.

**Table 6:** Pixel statistics before and after the training of the network and the samples in Figure 21.

	Ground Truth		Detected		TP	TN	FP	FN	Union	IOU
	P	N	P	N						
Before Training	308	2396	413	2291	241	2224	172	67	480	50.21%
Train On FP Only	308	2396	407	2297	240	2229	167	68	475	50.53%
Train On FN Only	308	2396	559	2145	303	2140	256	5	564	53.72%
After Training	308	2396	553	2151	302	2145	251	6	559	54.03%

P: positive; N: negative; TP: true positive; TN: true negative; FP: false positive; FN: false negative; IOU: intersection over union.

**Table 7:** Pixel statistics before and after the training of the network and the samples in Figure 22.

	Ground Truth		Detected		TP	TN	FP	FN	Union	IOU
	P	N	P	N						
Before Training	254	2450	324	2380	187	2313	137	67	391	47.83%
After Training	254	2450	379	2325	217	2288	162	37	416	52.16%

P: positive; N: negative; TP: true positive; TN: true negative; FP: false positive; FN: false negative; IOU: intersection over union.

**Table 8:** Pixel statistics before and after the training of the network and the samples in Figure 27.

	Ground Truth		Detected		TP	TN	FP	FN	Union	IOU
	P	N	P	N						
Before Training	298	726	331	693	201	596	130	97	428	46.96%
Train On FP Only	298	726	272	752	198	652	74	100	372	53.23%
Train On FN Only	298	726	355	669	218	589	137	80	435	50.11%
After Training	298	726	296	728	215	645	81	83	379	56.73%

P: positive; N: negative; TP: true positive; TN: true negative; FP: false positive; FN: false negative; IOU: intersection over union.

## Data Availability

Data are contained within the article.
